# Prognostic significance of microinvasion with ductal carcinoma in situ of the breast: a meta-analysis

**DOI:** 10.1007/s10549-022-06800-3

**Published:** 2022-11-24

**Authors:** Sho Shiino, Cecily Quinn, Graham Ball, Binafsha M. Syed, Sasagu Kurozumi, Hitoshi Tsuda, Emad A. Rakha

**Affiliations:** 1 Nottingham Breast Cancer Research Centre, Division of Cancer and Stem Cells, School of Medicine, University of Nottingham, Nottingham, UK; 2 Department of Breast Surgery, National Cancer Center Hospital, Tokyo, Japan; 3 Histopathology, Irish National Breast Screening Programme, St. Vincent’s University Hospital, Dublin, Ireland; 4 John Van Geest Cancer Research Centre, School of Science & Technology, Nottingham Trent University, Clifton Campus, Clifton Lane, Nottingham, UK; 5 Medical Research Centre, Liaquat University of Medical & Health Sciences, Jamshoro, Pakistan; 6 Department of Breast Surgery, International University of Health and Welfare, Narita, Japan; 7 Department of General Surgical Science, Gunma University Graduate School of Medicine, Maebashi, Japan; 8 Department of Basic Pathology, National Defense Medical College Hospital, Tokorozawa, Japan; 9 Department of Histopathology, Nottingham University Hospital NHS Trust, City Hospital Campus, Hucknall Road, Nottingham, NG5 1PB UK

**Keywords:** Breast cancer, Ductal carcinoma in situ with microinvasion, Ductal carcinoma in situ, Prognosis, Meta-analysis

## Abstract

**Purpose:**

Ductal carcinoma in situ (DCIS) associated with invasive carcinoma ≤ 1 mm in size is defined as DCIS with microinvasion (DCIS/microinvasion) rather than as invasive breast carcinoma. The number of patients with microinvasion accounts for < 1% of all breast cancer in published studies. As the numbers are limited, the prognostic significance of DCIS/microinvasion has not been clearly elucidated. This meta-analysis aimed to investigate the survival differences between patients with DCIS/microinvasion and those with pure DCIS.

**Methods:**

A meta-analysis following the Preferred Reporting Items for Systematic Reviews and Meta-Analyses (PRISMA) methodology was performed. We searched three electronic databases (MEDLINE, Cochrane Library, and EMBASE) and included observational studies published in English that contained survival details of patients with either DCIS or DCIS/microinvasion.

**Results:**

This study identified 26 studies that described the clinicopathological characteristics of patients in both the DCIS and DCIS/microinvasion groups. Survival differences were evaluated in 10 of 26 studies. Disease-free survival and loco-regional recurrence-free survival were significantly shorter in patients with DCIS/microinvasion than in those with DCIS (Hazard ratio, 1.52; 95% confidence interval, 1.11–2.08; *p* = 0.01 and hazard ratio, 2.53; 95% confidence interval, 1.45–4.41; *p* = 0.001, respectively). Both overall survival and distant metastasis-free survival tended to be shorter in patients with DCIS/microinvasion than in patients with DCIS (Hazard ratio, 1.63; 95% CI, 0.63–4.23; *p* = 0.31 and hazard ratio, 1.85; 95% confidence interval, 0.74–4.66; *p* = 0.19, respectively) but the difference was not statistically significant.

**Conclusion:**

Our meta-analysis suggests that DCIS/microinvasion may display more aggressive biological and clinical behavior than pure DCIS, highlighting the potential need for closer follow-up and consideration of adjuvant treatment strategies in DCIS patients with microinvasive disease.

**Supplementary Information:**

The online version contains supplementary material available at 10.1007/s10549-022-06800-3.

## Introduction

Microinvasive carcinoma, which is defined as invasive breast carcinoma ≤ 1 mm in size (microinvasion) [1, 2], is the earliest morphologically recognized form of invasive breast carcinoma (IBC). Microinvasion is usually observed in association with ductal carcinoma in situ (DCIS) and the term DCIS with microinvasion (DCIS-Mi) is frequently used by pathologists. Although the diagnosis of pure DCIS is common, primarily due to the impact of population based mammographic screening programs, and accounts for approximately 20% of all breast cancer diagnoses [3], the diagnosis of DCIS-Mi accounts for < 1% of cases [4].

While some investigative studies have reported survival differences between the DCIS-Mi and DCIS groups [5, 6], others have observed similar survival rates in the two groups [7, 8]. Therefore, the prognostic significance of DCIS-Mi and its biological significance compared with pure DCIS are not fully elucidated and clinicians are uncertain regarding the metastatic risks and the potential benefits of adjuvant treatment strategies. Knowledge of the survival differences between patients with DCIS-Mi compared with those with pure DCIS would enhance our knowledge of the biology of this disease and potentially assist decision making regarding adjuvant treatment plans. A randomized controlled trial or a single observational study to investigate survival differences between DCIS-Mi and DCIS patients would be difficult due to the relative rarity of DCIS-Mi. For this reason, a meta-analytic approach is suitable to determine survival differences.

The study aim was to conduct a meta-analysis to investigate the survival differences between DCIS-Mi and DCIS and assess the impact of clinicopathological characteristics on survival of patients with DCIS-Mi.

## Methods

### Meta-analysis registration

We used Preferred Reporting Items for Systematic review and meta-analysis protocols (PRISMA-P, 2015) to ensure transparent and complete reporting of this research [9] (Supplementary Table S1). We described the PICO elements (participants, interventions, comparators, and outcomes), primary/secondary endpoints, inclusion/exclusion criteria, and subgroup analysis for the clinical question as shown in Supplementary Table S2. We prospectively registered our protocol on PROSPERO, which is an international prospective register of systematic reviews (registration number: CRD42020163096, available from: https://www.crd.york.ac.uk/prospero/display_record.php?ID=CRD42020163096). Amendments of the published first protocol are available on the same site.

### Search strategy

Following registration of our protocol on PROSPERO, we searched the following three electronic databases: MEDLINE, Cochrane Library, and EMBASE. Each search strategy for electronic databases is shown in Supplementary Table S3. We consulted with an experienced searcher (AA) “see acknowledgment” to confirm the validity of this search strategy. We merged the three search results using JabRef software (https://www.jabref.org/) and Microsoft Excel software ver.16.40.

### Eligibility criteria of articles

**Fig. 1:**
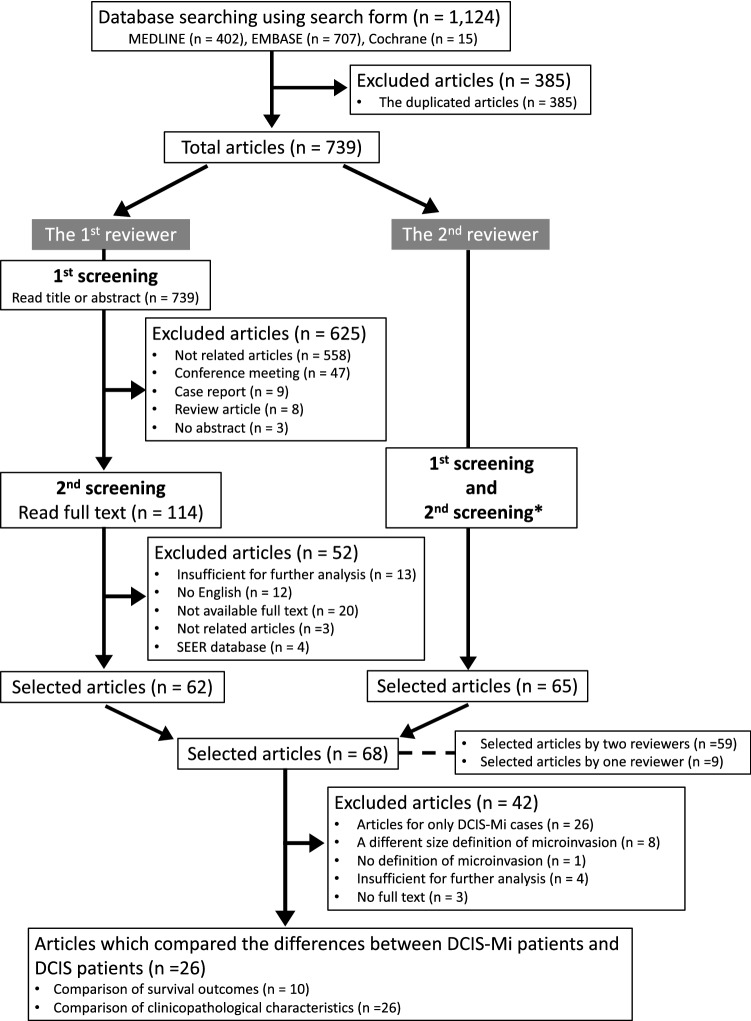
Flow chart of the selection procedure used in this meta-analysis. *DCIS*, ductal carcinoma in situ; *DCIS-Mi* ductal carcinoma in situ with microinvasion; *SEER* the Surveillance, Epidemiology, and End Results *Details were shown in Supplementary Figure S1

In our meta-analysis, we included observational studies that compared differences in survival between patients with DCIS-Mi (DCIS-Mi group: presence of microinvasion) and those with DCIS (DCIS group: no invasion). The first screening was then performed by reading titles or abstracts. The second screening was performed by performing a full-text review of each article (Fig. 1 and Supplementary Figure S1). In the final selected articles, we consulted with each corresponding author via email to retrieve sufficient data to perform our meta-analysis.

The processes of article selection from the three databases were independently performed by two authors (SS and BMS). Discrepancies between author assessments of articles were discussed until a consensus was reached. Any discrepancies of selected articles between the two independent reviewers were evaluated by using the kappa (κ) coefficient.

### Endpoints and data extraction

The primary and secondary endpoints of our meta-analysis were disease-free survival (DFS) and overall survival (OS), respectively, (Supplementary Table S2). Timing was not restricted. We performed subgroup analysis regarding the loco-regional recurrence-free survival (LRFS), distant metastasis-free survival (DMFS), and risk of bias group. We also evaluated the effect of each clinicopathological characteristic on survival of patients with DCIS-Mi.

Clinicopathological information was extracted from the finally selected articles. DCIS-Mi was defined as DCIS with invasive carcinoma measuring ≤ 1 mm in maximum dimension [1, 2, 10]. The TNM classification was used to define the lymph node (LN) status [10]. Nuclear grade (NG) was classified according to either the Van Nuys classification [11] or the Nottingham grading system [12, 13]. Estrogen receptor (ER) and progesterone receptor (PR) status, determined using immunohistochemical (IHC) staining, were considered positive using a cutoff of ≥ 1% in reference to the American Society of Clinical Oncology/College American Pathologists (ASCO/CAP) guideline [14]. Human epidermal growth factor receptor 2 (HER2) expression level was defined as positive with reference to the ASCO/CAP guidelines at the time of publication [15–17].

### Quality assessment

All selected articles were evaluated by two independent authors (SS, SK) by using the Risk of Bias Assessment tool for non-randomized Studies (RoBANS) which includes the following six domains: (1) selection of participants, (2) confounding variables, (3) measurement of exposure, (4) blinding of outcome assessments, (5) incomplete outcome data, and (6) selective outcome reporting [18]. Disagreements between the reviewers were discussed until consensus was reached. Differences in the quality assessments between the two independent reviewers were assessed by calculating the κ coefficient.

### Statistical analysis

Hazard ratios (HRs) and 95% confidence intervals (CIs) were extracted from each selected article. The meta-analysis was conducted using a fixed-effects model to minimize heterogeneity among the extracted studies. Heterogeneity was also evaluated using a random-effects model. Statistical heterogeneity was assessed by performing the I^2^ test and categorized according to the following definitions: > 50%, high heterogeneity; 25–50%, moderate heterogeneity; and 0–25%, low heterogeneity. Forest plots were used to visualize the heterogeneity. Funnel plots were constructed to evaluate publication bias. A two-sided *p*-value of > 0.05 was considered to be indicative of statistical significance. For I^2^ values > 50% or significant, either sensitivity analysis or meta-regression analysis was performed to determine the reasons for the high heterogeneity. The meta-analysis, risk of bias graph, and bias summary were performed by using Review Manager (RevMan) version 5.3 [19].

When HR data or survival data were not explicitly stated in the literature, cumulative survival values were extracted from the relevant Kaplan–Meier survival curves by using Engauge Digitizer software v12. HR values were estimated from the extracted cumulative survival values by using a Microsoft Excel spreadsheet reported previously [20]. Meta-regression analysis was performed to determine whether the values of any clinicopathological factors were associated with the effect size.

κ value results were defined as follows: > 0.75, excellent agreement; 0.40–0.75, fair to good agreement; and < 0.40, poor agreement [21]. A two-sided Wilcoxon signed-rank test was used to compare the two paired rates for each clinicopathological factor between the DCIS-Mi and DCIS groups. We calculated κ value and performed a two-sided Wilcoxon signed-rank test using SPSS version 27 (IBM SPSS, Armonk, NY, USA).

## Results

### Results of search strategy

The two reviewers independently evaluated all selected 739 articles using a search strategy for the first and second screenings, wherein 68 articles remained (Fig. 1 and Supplementary Figure S1). The discordance rate of the selected articles between the two reviewers was 1.2% (9/739). The κ value was 0.922, which represented excellent agreement. Of the 68 articles, we excluded articles which described only DCIS-Mi cases (*n* = 26), used a different size definition or no definition of microinvasion (*n* = 9), studies with data insufficient for further statistical analysis (*n* = 4) and no full text (*n* = 3). The remaining 26 articles documented the clinicopathological characteristics of patients in the two groups, presented in Supplementary Table S4. Ten of the 26 studies described the actual survival differences (either DFS or OS) between DCIS-Mi and DCIS [5, 7, 8, 22–28] (Table 1).

**Table 1:** The main characteristics of the studies with survival outcomes used in the meta-analysis

Author	Published year	Country	Trial method	Study term	Inclusion criteria	Exclusion criteria	Study endpoint	Total number cases (*n*)	Pathological review for DCIS-Mi
Fang Y, et al	2016	China	Retrospective study	2002–2014	DCIS, DCIS-Mi, or DCIS-T1a	NA	NA	DCIS: 359, DCIS-Mi: 84 (DCIS-T1a: 159)	NA
Wang L, et al	2015	China	Retrospective study	2002–2009	DCIS or DCIS-Mi	NA	NA	DCIS: 451, DCIS-Mi: 131	All patients who were diagnosed as DCIS-Mi, were confirmed by two of the authors
Yu KD, et al	2011	China	Retrospective study	1998–2007	Female, primary breast cancer without distant metastases, diagnosis of breast carcinoma in situ (pure DCIS, DCIS-Mi or DCIS with invasion)	NA	NA	DCIS: 271, DCIS-Mi: 67, DCIS-invasive component: 212	The pathologic and IHC outcomes were originally checked and approved by two pathologists
Parikh RP, et al	2012	USA	Retrospective study	1973–2004	DCIS or DCIS-Mi	NA	LRFS, DMFS, OS	DCIS: 321, DCIS-Mi: 72	NA
Pu T, et al	2018	China^*1^	Retrospective study	1997–2014	DCIS or DCIS-Mi or IDC (T1)	NA	Primary: DFS, Secondary: BCSS^*2^	DCIS: 280, DCIS-Mi: 242, IDC (T1): 347	All diagnoses were confirmed by two pathologists
Kim M, et al	2018	Korea	Retrospective study	2003–2014	DCIS or DCIS-Mi	NA	NA	DCIS: 477, DCIS-Mi: 136	All cases were reviewed by two pathologists
Sue G, et al	2013	USA	Retrospective study	2000–2003	DCIS or DCIS-Mi	Invasive carcinoma which is greater than 1 mm	Loco-regional recurrence and/or distant metastasis, OS	DCIS:154, DCIS-Mi: 51	All cases were reviewed by a board-certified pathologist at their institution
Bertozzi S, et al	2019	Italy	Retrospective study	2002–2016	DCIS or IDC (sized ≤ 2 cm)	All histotypes other than ductal carcinoma, male breast cancer, and tumors sized > 2 cm	NA	DCIS: 543, DCIS-Mi: 84, IDC (T1): 2111	NA
Mamtani A, et al	2019	USA^*1^	Retrospective study	1995–2015	DCIS or DCIS-Mi	NA	LRR	DCIS: 2700, DCIS-Mi: 421	NA
Zheng J, et al	2020	China	Retrospective study	2014–2018	DCIS or DCIS-Mi or IDC (T1)	NA	NA	DCIS: 308, DCIS-Mi: 92, IDC (T1a/b/c): 1486	All cases were reviewed by two senior pathologists

*BCSS* breast cancer specific survival, *DCIS* ductal carcinoma in situ, *DCIS-Mi* ductal carcinoma in situ with microinvasion, *DFS* disease-free survival, *DMFS* distant metastasis-free survival, *IDC* invasive ductal carcinoma, *IHC* immunohistochemical, *LRFS* loco-regional recurrence-free survival, *LRR* loco-regional recurrence, *NA* not available, *OS* overall survival

^*^^1^Multi-institutions

^*^^2^BCSS: The time from the date of diagnosis to death of breast cancer

In two studies [8, 24], DFS was reported as recurrence-free survival (RFS: from the date of diagnosis of the primary tumor to the date of the earliest local, regional or distant relapse or contralateral breast cancer) and both of which are also included in our DFS meta-analysis. The RoBANS tool was used to evaluate the 10 selected articles (Supplementary Figure S2A and S2B). In each domain group, κ values for the concordance between the two reviewers are as shown in Supplementary Figure S2C.

### Differences in the effects on DFS in patients with DCIS-Mi

**Fig. 2:**
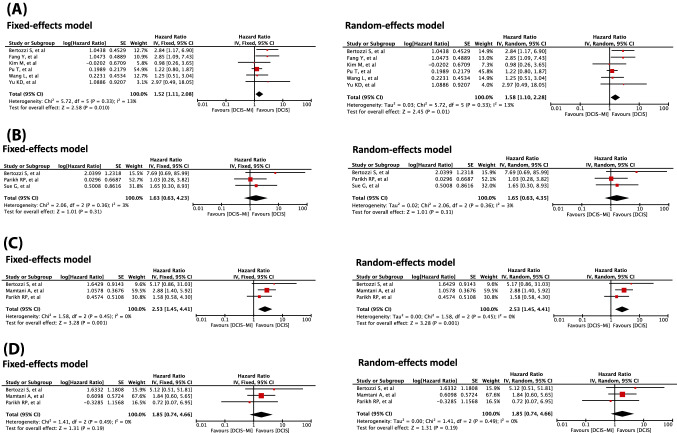
Forest plots comparing the patients’ survival between the DCIS-Mi group and DCIS group *DCIS* ductal carcinoma in situ, *DCIS-Mi* ductal carcinoma in situ with microinvasion, *DFS* disease-free survival, *DMFS* distant metastasis-free survival, *LRFS* loco-regional recurrence-free survival, *OS* overall survival. **A** Comparison of DFS between the DCIS-Mi and DCIS groups. **B** Comparison of OS between the DCIS-Mi and DCIS groups. **C** Comparison of LRFS between the DCIS-Mi and DCIS groups. **D** Comparison of DMFS between the DCIS-Mi and DCIS groups

Five studies evaluated DFS [5, 22, 23, 26, 28] and two studies evaluated RFS [8, 24] in patients with DCIS-Mi compared to those with pure DCIS, producing a total of seven studies eligible for DFS analysis. We were unable to calculate the 95% CI by using specialized software in one of these studies [28] due to extremely wide 95% CIs. Following exclusion of this study, there were a total of 744 patients in the DCIS-Mi group and 2381 patients in the DCIS group (Supplementary Table S5). Our meta-analysis revealed that DFS was significantly shorter for DCIS-Mi than for DCIS [(Fixed-effects model) HR, 1.52; 95% CI, 1.11–2.08; *p* = 0.01, (Random-effects model) HR, 1.58; 95% CI, 1.10–2.28; *p* = 0.01] (Fig. 2A). Significant heterogeneity was not observed in the analyses [(Fixed-effects model) *I*^*2*^ = 13%; *p* = 0.33 (Random-effects model) *I*^*2*^ = 13%; *p* = 0.33].

### Differences in the effects on OS in patients with DCIS-Mi

A total of six articles compared OS between DCIS and DCIS-Mi [5, 7, 22, 25, 26, 28]. We were unable to calculate the 95% CI in three of these studies by our method due to wide 95% CIs [5, 22, 28]. Following exclusion of these studies, the total numbers of patients were 207 in the DCIS-Mi group and 1,018 in the DCIS group (Supplementary Table S5). Our meta-analysis showed that OS tended to be shorter in the DCIS-Mi group than in the DCIS group, but the difference was not significant [(Fixed-effects model) HR, 1.63; 95% CI, 0.63–4.23; *p* = 0.31, (Random-effects model) HR, 1.65; 95% CI, 0.63–4.35; *p* = 0.31] (Fig. 2B). Significant heterogeneity was not observed in the analyses [(Fixed-effects model) *I*^*2*^ = 3%; *p* = 0.36, (Random-effects model) *I*^*2*^ = 3%; *p* = 0.36].

### Subgroup analyses of the DFS events (LRFS and DMFS)

We compared the LRFS differences between the DCIS group and DCIS-Mi group. Three studies were included in the analysis [7, 26, 27]. One of those studies which has reported the cumulative incidence of loco-regional recurrence is also included in our LRFS meta-analysis [27]. The total numbers of patients were 577 in the DCIS-Mi group and 3,564 in the DCIS group (Supplementary Table S5). The meta-analysis showed that LRFS was significantly shorter in the DCIS-Mi group than in the DCIS group [(Fixed-effects model) HR, 2.53; 95% CI, 1.45–4.41; *p* = 0.001, (Random-effects model) HR, 2.53; 95% CI, 1.45–4.41; *p* = 0.001] (Fig. 2C). Heterogeneity was not observed in the analyses [(Fixed-effects model) *I*^*2*^ = 0%; *p* = 0.45, (Random-effects model) *I*^*2*^ = 0%; *p* = 0.45].

We also compared the DMFS differences between the DCIS group and DCIS-Mi group. Three studies were included in this analysis [7, 26, 27]. The total numbers of patients were 577 in the DCIS-Mi group and 3,564 in the DCIS group (Supplementary Table S5). No significant differences were observed between the two groups [(Fixed-effects model) HR, 1.85; 95% CI, 0.74–4.66; *p* = 0.19, (Random-effects model) HR, 1.85; 95% CI, 0.74–4.66; *p* = 0.19] (Fig. 2D). Heterogeneity was not observed in the analyses [(Fixed-effects model) *I*^*2*^ = 0%; *p* = 0.49, (Random-effects model) *I*^*2*^ = 0%; *p* = 0.49].

### Subgroup analysis based on the assessment of risk of bias

In the subgroup analysis of low-risk of bias group, the DCIS-Mi group had significantly shorter DFS than the DCIS group [(Fixed-effects model) HR, 1.61; 95% CI, 1.14–2.29; *p* = 0.008, (Random-effects model) HR, 1.94; 95% CI, 1.11–3.37; *p* = 0.02]. However, such differences became insignificant in the subgroup analysis of high-risk of bias group [(Fixed-effects model) HR, 1.16; 95% CI, 0.55–2.42; *p* = 0.70, (Random-effects model) HR, 1.16; 95% CI, 0.55–2.42; *p* = 0.70] (Supplementary Figure S3A). There was no significant difference in OS between two groups in both low-risk of bias group and high-risk of bias group (Supplementary Figure S3B).

### Effect of each clinicopathological characteristic on DFS in DCIS-Mi patients

In the articles included, DFS analysis for patients with DCIS-Mi who did not receive adjuvant therapy (hormone therapy and/or chemotherapy) had been performed in only two studies [5, 23]. We assessed the effect of each clinicopathological factor on survival in the DCIS-Mi group to evaluate the natural history of DCIS-Mi (Supplementary Figure S4).

There were no significant differences between the effects of age < 50 and ≥ 50 years [Supplementary Figure S4A: (Fixed-effects model) HR, 2.75; 95% CI, 0.50–15.22; *p* = 0.25, (Random-effects model) HR, 3.28; 95% CI, 0.10–112.85; *p* = 0.51] or between the effects of ER-positivity and ER-negativity on DFS in the DCIS-Mi patients [Supplementary Figure S4B: (Fixed-effects model) HR, 0.38; 95% CI, 0.07–1.97; *p* = 0.25, (Random-effects model) HR, 0.38; 95% CI, 0.07–1.97; *p* = 0.25].

Meanwhile, DFS was significantly longer in DCIS-Mi patients who were PR positive than in those who were PR negative [Supplementary Figure S4C: (Fixed-effects model) HR, 0.17; 95% CI, 0.03–0.95; *p* = 0.04, (Random-effects model) HR, 0.17; 95% CI, 0.03–0.95; *p* = 0.04]. DFS tended to be shorter in DCIS-Mi patients who were HER2 positive than in those who were HER2 negative [Supplementary Figure S4D: (Fixed-effects model) HR, 5.79; 95% CI, 0.99–33.90; *p* = 0.05, (Random-effects model) HR, 5.99; 95% CI, 0.52–69.75; *p* = 0.15], but the difference was not significant.

We were unable to extract the differences between the effects of NG3 versus NG1 or 2, or axillary LN-positivity versus LN-negativity on DFS of DCIS-Mi patients who had not received adjuvant treatment. One study that evaluated DFS in patients with DCIS-Mi with a single versus multiple foci of microinvasive carcinoma, observed no survival difference between these two groups (HR, 0.66; 95% CI, 0.05–9.09; *p* = 0.754) [5].

### Clinicopathological characteristics of selected articles

In the 26 studies detailed in Supplementary Table S4, the median number of patients was 58 (range: 12–421) for the DCIS-Mi group and 258 (range: 44–2721) for the DCIS group. We compared the rate of each clinicopathological characteristic between the DCIS-Mi group and DCIS group using paired results. The median rates of each clinicopathological characteristic are presented in Supplementary Table S6. Total lesion size > 2 cm (*p* = 0.046), axillary LN metastasis (*p* < 0.001), comedo necrosis (*p* = 0.005), NG3 (*p* = 0.001), HER2-positivity (*p* = 0.018), and adjuvant chemotherapy (*p* = 0.043) were significantly higher in the DCIS-Mi group than in the DCIS group. ER-positivity (*p* = 0.028) and PR-positivity (*p* = 0.028) were significantly lower in the DCIS-Mi group.

### Evaluation of the influences of each clinicopathological characteristic on the hazard ratio for DFS analysis

Meta-regression analysis was performed to investigate if the rate of each clinicopathological factor in the DCIS-Mi group was associated with the HR of DFS analysis. None of these factors (premenopausal status, total lesion size of > 2 cm, axillary LN metastasis, NG3, ER-positivity, PR-positivity, HER2-positivity, and the use of the adjuvant treatment; hormone therapy, chemotherapy, or radiotherapy) significantly influenced the HR of DFS analysis (Supplementary Table S7).

### Evaluation of publication bias

We were unable to evaluate the risks of publication bias by statistical analysis because each analysis contained < 10 studies. Funnel plots of DFS and OS analysis are summarized in Supplementary Figure S5.

## Discussion

In our meta-analysis, we demonstrated that DFS and LRFS were significantly shorter in the DCIS-Mi group than in the DCIS group. Meanwhile, the differences in OS and DMFS were not significant. Some studies that have reported differences in survival between DCIS-Mi and DCIS groups, as determined from the Surveillance, Epidemiology, and End Results (SEER) database [6, 29, 30] showed that DCIS-Mi was significantly associated with shorter survival. However, it is difficult to draw firm conclusions from these reports as the SEER database contains multi-institutional data with variations in the definition of microinvasive carcinoma applied. In contrast, we aimed to select only studies that utilized the now standardized definition of microinvasive carcinoma as invasive carcinoma ≤ 1 mm.

In our analysis of selected articles, larger lesion size, axillary LN metastasis, comedo necrosis, NG3, ER-negativity, PR-negativity, and HER2-positivity were significantly more frequently observed in association with DCIS-Mi than with pure DCIS. Results using the SEER database showed that DCIS-Mi was more likely to be ER negative, PR negative, HER2 positive, high NG, and high LN stage compared with the pure DCIS group [6]. The results of our meta-analysis are similar and support the view that DCIS-Mi is a biologically more aggressive disease than pure DCIS.

However, it is unclear whether microinvasion impact on patient outcome is related to the microinvasive disease or to the fact that microinvasion is often associated with high-risk DCIS which may account for any observed poor outcome. Therefore, we also investigated the effect of each clinicopathological factor on survival in DCIS-Mi patients in an attempt to further interrogate the biology and natural history of this disease. PR-positivity was significantly associated with longer DFS and HER2-positivity was marginally associated with shorter DFS in the patients with DCIS-Mi. Some authors have reported that adjuvant treatment for small HER2 positive breast cancer could have survival benefit [31–33]. However, there is still debate as to whether adjuvant chemotherapy with or without trastuzumab is necessary for the DCIS-Mi group. The combination of survival data and the profile and impact of clinicopathological characteristics suggest that adjuvant treatment strategies could be considered in the management of patients with DCIS-Mi although our selected articles for those analyses were limited in only two studies (Supplementary Figure S4). Further investigations will be required to validate this proposal.

We were unable to evaluate the influence of differing axillary LN status on survival in the DCIS-Mi group. A meta-analysis of sentinel lymph node (SLN) biopsy findings in patients with microinvasive carcinoma previously reported rates of 3.2, 4.0, and 2.9% for macrometastasis, micrometastasis, and isolated tumor cells, respectively [34]. Some previous studies have also reported on the frequency of axillary LN metastases in DCIS-Mi patients [35, 36]. However, the influence of such axillary LN metastases on survival difference between DCIS-Mi patients and DCIS patients is currently unclear. Some studies have observed [37, 38] no difference in local recurrence rates in patients with a single versus multiple foci of microinvasion but did not report on DFS or OS.

DCIS is a recognized precursor of IBC [39, 40]. At genomic level, DCIS with adjacent invasive carcinoma displays a more aggressive profile than pure DCIS [41]. However, genetic characteristics were not examined in our meta-analysis.

This study has some limitations that should be considered when interpreting our results. Firstly, in published studies that did not include HR or 95% CI data, we calculated those data using specialized software. This technique is commonly utilized in meta-analyses but may result in discrepancies between original and calculated data. Additionally, we could not extract 95% CI for four of the studies (DFS analysis: one study, OS analysis: three studies) using this software and results for OS analysis, in particular, may be insufficient to draw reliable conclusions. Secondly, we were unable to fully evaluate the risk ratio adjusted for some clinicopathological factors including race, type of primary surgery, and the administration of adjuvant treatments. However, we did verify that the rates of several clinicopathological characteristics (lesion size, axillary LN status, NG, ER, PR, and HER2 status) did not affect the risk ratio in each study using meta-regression analysis. Thirdly, some of the studies did not specify if the pathological variables such as NG, ER, PR, or HER2 pertained to the DCIS or the microinvasive carcinoma component. However, it is recognized that the concordance between DCIS and the co-existing invasive carcinoma is relatively high [42, 43].

In conclusion, our meta-analysis demonstrates that patients with DCIS-Mi have shorter DFS or LRFS than those with pure DCIS, suggesting a more locally/regionally aggressive natural history for DCIS patients with microinvasive disease. DCIS-Mi also appears to have a more aggressive biological phenotype with a greater tendency toward larger lesion size, axillary LN metastases, higher grade, comedo necrosis, ER-negativity, PR-negativity, and HER2-positivity. The overall findings suggest that patients with DCIS-Mi may require closer follow-up compared to patients with pure DCIS and that adjuvant treatment strategies may need to be considered in patients with DCIS-Mi, particularly if associated with more aggressive biological indices.

## Supplementary Information

Below is the link to the electronic supplementary material.

Supplementary file1 (DOCX 2227 kb)

## Data Availability

Enquiries about data availability should be directed to the authors.

## Abbreviations

DCIS: Ductal carcinoma in situ
DCIS-Mi: Ductal carcinoma in situ with microinvasion
PRISMA-P: Preferred reporting items for systematic review and meta-analysis protocols
PICO: Participants, interventions, comparators, and outcomes
PROSPERO: A prospective international register of systematic reviews
DFS: Disease-free survival
OS: Overall survival
LRFS: Loco-regional recurrence-free survival
DMFS: Distant metastasis-free survival
LN: Lymph node
NG: Nuclear grade
ER: Estrogen receptor
PR: Progesterone receptor
IHC: Immunohistochemical
ASCO/CAP: American Society of Clinical Oncology/College American Pathologists
HER2: Human epidermal growth factor receptor 2
RoBANS: Risk of bias assessment tool for non-randomized Studies
HR: Hazard ratio
CI: Confidence interval
RFS: Recurrence-free survival
SEER: Surveillance, Epidemiology, and End Results
SLN: Sentinel lymph node
IBC: Invasive breast carcinoma
