# Supplementary material for: Prognostic significance of microinvasion with ductal carcinoma in situ of the breast: a meta-analysis
Source: Breast Cancer Res Treat. 2022 Nov 24;197(2):245–54. doi: 10.1007/s10549-022-06800-3 (PMC9823049; doi:10.1007/s10549-022-06800-3)
Supplement: Supplementary file 1 — Supplementary file1 (DOCX 2227 kb) [file 10549_2022_6800_MOESM1_ESM.docx]

**Supplementary Table S1.**

PRISMA-P 2015 checklist

| **Section/topic** | **#** | **Checklist item** | **Information reported** | | **Line number(s)** |
| --- | --- | --- | --- | --- | --- |
|  |  |  | **Yes** | **No** |  |
| **ADMINISTRATIVE INFORMATION** | | | | | |
| **Title** | | | | | |
| Identification | 1a | Identify the report as a protocol of a systematic review |  |  | Described in the title |
| Update | 1b | If the protocol is for an update of a previous systematic review, identify as such |  |  |  |
| **Registration** | 2 | If registered, provide the name of the registry (e.g., PROSPERO) and registration number in the Abstract |  |  | Described in the “Methods” section |
| **Authors** | | | | | |
| Contact | 3a | Provide name, institutional affiliation, and e-mail address of all protocol authors; provide physical mailing address of corresponding author |  |  | Described on the first page |
| Contributions | 3b | Describe contributions of protocol authors and identify the guarantor of the review |  |  | Described in the “Author Contributions” section |
| **Amendments** | 4 | If the protocol represents an amendment of a previously completed or published protocol, identify as such and list changes; otherwise, state plan for documenting important protocol amendments |  |  | Described in the “Methods” section |
| **Support** | | | | | |
| Sources | 5a | Indicate sources of financial or other support for the review |  |  | Described in the “Funding” section |
| Sponsor | 5b | Provide name for the review funder and/or sponsor |  |  |  |
| Role of sponsor/funder | 5c | Describe roles of funder(s), sponsor(s), and/or institution(s), if any, in developing the protocol |  |  |  |
| **INTRODUCTION** | | | | | |
| **Rationale** | 6 | Describe the rationale for the review in the context of what is already known |  |  | Described in the “Introduction” section |
| **Objectives** | 7 | Provide an explicit statement of the question(s) the review will address with reference to participants, interventions, comparators, and outcomes (PICO) |  |  | Supplementary Table 2 |
| **METHODS** | | | | | |
| **Eligibility criteria** | 8 | Specify the study characteristics (e.g., PICO, study design, setting, time frame) and report characteristics (e.g., years considered, language, publication status) to be used as criteria for eligibility for the review |  |  | Supplementary Table 2 |
| **Information sources** | 9 | Describe all intended information sources (e.g., electronic databases, contact with study authors, trial registers, or other grey literature sources) with planned dates of coverage |  |  | Described in the “Methods” section, Supplementary Table 2 |
| **Search strategy** | 10 | Present draft of search strategy to be used for at least one electronic database, including planned limits, such that it could be repeated |  |  | Supplementary Table 3 |

| **Section/topic** | **#** | **Checklist item** | **Information reported** | | **Line number(s)** |
| --- | --- | --- | --- | --- | --- |
|  |  |  | **Yes** | **No** |  |
| ***STUDY RECORDS*** | | | | | |
| Data management | 11a | Describe the mechanism(s) that will be used to manage records and data throughout the review |  |  | Described in the “Methods” section |
| Selection process | 11b | State the process that will be used for selecting studies (e.g., two independent reviewers) through each phase of the review (i.e., screening, eligibility, and inclusion in meta-analysis) |  |  | Described in the “Methods” section, Figure 1, and Supplementary Figure 1 |
| Data collection process | 11c | Describe planned method of extracting data from reports (e.g., piloting forms, done independently, in duplicate), any processes for obtaining and confirming data from investigators |  |  | Described in the “Methods” section, Supplementary Table 2 |
| **Data items** | 12 | List and define all variables for which data will be sought (e.g., PICO items, funding sources), any pre-planned data assumptions and simplifications |  |  | Described in the “Methods” section, Supplementary Table 2 |
| **Outcomes and prioritization** | 13 | List and define all outcomes for which data will be sought, including prioritization of main and additional outcomes, with rationale |  |  | Described in the “Methods” section, Supplementary Table 2 |
| **Risk of bias in individual studies** | 14 | Describe anticipated methods for assessing risk of bias of individual studies, including whether this will be done at the outcome or study level, or both; state how this information will be used in data synthesis |  |  | Described in the “Methods” section, Supplementary Figure 2 |
| ***DATA*** | | | | | |
| **Synthesis** | 15a | Describe criteria under which study data will be quantitatively synthesized |  |  | Described in the “Methods” section |
|  | 15b | If data are appropriate for quantitative synthesis, describe planned summary measures, methods of handling data, and methods of combining data from studies, including any planned exploration of consistency (e.g., *I* ^2^, Kendall’s tau) |  |  | Described in the “Methods” section |
|  | 15c | Describe any proposed additional analyses (e.g., sensitivity or subgroup analyses, meta-regression) |  |  | Described in the “Methods” section |
|  | 15d | If quantitative synthesis is not appropriate, describe the type of summary planned |  |  |  |
| **Meta-bias(es)** | 16 | Specify any planned assessment of meta-bias(es) (e.g., publication bias across studies, selective reporting within studies) |  |  | Described in the “Methods” section |
| **Confidence in cumulative evidence** | 17 | Describe how the strength of the body of evidence will be assessed (e.g., GRADE) |  |  | Supplementary Figure 2 |

**Supplementary Table S2.**

Protocol outline registered on PROSPERO

| **Type and method of review** | Meta-analysis | |
| --- | --- | --- |
| **Clinical question** | How does ductal carcinoma *in situ* with microinvasion (DCIS-Mi) of the breast affect survival in patients compared to DCIS? | |
| **PICO** | **P: Participants** | Patients with DCIS-Mi or DCIS |
|  | **I: Interventions** | Patients with DCIS-Mi |
|  | **C: Comparators** | Patients with DCIS |
|  | **O: Outcomes** | **Main outcome:** DFS (disease-free survival: from the diagnostic date of primary tumors to the earliest of all recurrences, second cancer, or death)  **Additional outcome:** OS (overall survival: from the diagnostic date of primary tumors to the date of death or last follow-up) |
| **Endpoint** | **Primary** | DFS (disease-free survival: from the diagnostic date of primary tumors to the earliest of all recurrences, second cancer, or death) |
|  | **Secondary** | OS (overall survival: from the diagnostic date of primary tumors to the date of death or last follow-up) |
| **Searches** | The three electronic databases   1. MEDLINE 2. Cochrane Library 3. EMBASE | |
| **Inclusion criteria** | Studies that performed survival analysis between patients with DCIS-Mi and DCIS in the primary breast tumor. Only studies published in English will be included.  **Timing:** Not restricted | |
| **Exclusion criteria** | Duplicated articles | |
|  | **1^st^ screening**  **(Read title or abstract)** | 1. Article titles or abstracts that were unrelated to the topic 2. Conference meeting abstract or publication 3. Systematic review 4. Case report 5. Full title or abstract was unavailable 6. No abstract in English |
|  | **2^nd^ screening**  **(Read full-text)** | 1. Unrelated to our topic 2. Unavailable for further statistical analysis 3. Unavailable full text 4. English text was unavailable |

| **Data extraction** | **General information** | Author names  Publication year  Study design  Country  Total cases  Study term  Inclusion/exclusion criteria  Primary/secondary endpoint  Total cases  Definition of DCIS-Mi  Definition of hormone receptor/HER2 positivity |
| --- | --- | --- |
|  | **Clinicopathological characteristics** | Age  Menopausal status  Tumor size  Lymph node status  Presence of comedo necrosis  Histologic type (DCIS-Mi or DCIS)  Histologic grade or nuclear grade  Number of invasive foci  Primary surgery  Margin status  Hormone receptor status (ER and PR)  HER2 status  Ki-67 labeling index  Adjuvant treatment details (hormone therapy/chemotherapy/HER2-targeted therapy/radiotherapy) |
|  | **Outcome data** | DFS  OS  Kaplan–Meier curves  Hazard ratio and 95%CI |
| **Subgroup analysis** | | 1. LRFS; the time from the diagnostic date of primary tumors to the date of local recurrence, including the ipsilateral chest wall and regional lymph node 2. DMFS; the time from the diagnostic date of primary tumors to the date of distant metastasis 3. Risk of bias   a) High risk of bias: Articles which have more than one high-risk of bias in the six domains.  b) Low-risk of bias: Articles which have no high-risk of bias or one high-risk of bias in the six domains. |

Abbreviations: CI, confidential interval; DCIS, ductal carcinoma *in situ*; DCIS-Mi, ductal carcinoma *in situ* with microinvasion; DFS, disease-free survival; DMFS, distant metastasis-free survival; ER, estrogen receptor; HER2, human epidermal growth factor receptor 2; LRFS, loco-regional recurrence-free survival; OS, overall survival; PR, progesterone receptor

**Supplementary Table S3.**

Search strategies used in different electronic databases

MEDLINE

|  | **Formula** | **Results (n)** |
| --- | --- | --- |
| 1 | Exp Breast Neoplasms/ | 286648 |
| 2 | ((breast or mammary) adj2 (cancer* or neoplasm* or carcinoma* or malignan* or tumor* or tumour*)).mp. | 394798 |
| 3 | ((breast or mammary) and "invasive ductal carcinoma").mp. | 3775 |
| 4 | Exp Carcinoma, Ductal, Breast/ | 15533 |
| 5 | 1 or 2 or 3 or 4 | 394995 |
| 6 | (microinvasive or microinvasion or T1mi or "micro invasion" or "micro invasive").mp. | 2713 |
| 7 | Exp Carcinoma, Intraductal, Noninfiltrating/ | 10002 |
| 8 | Exp Carcinoma in Situ/ | 35606 |
| 9 | ("ductal carcinoma in situ" or DCIS or Tis or "intraductal carcinoma").mp. | 11361 |
| 10 | 7 or 8 or 9 | 41900 |
| 11 | 5 and 6 and 10 | 402 |

EMBASE

|  | **Formula** | **Results (n)** |
| --- | --- | --- |
| 1 | Exp breast tumor/ | 517082 |
| 2 | ((breast or mammary) adj2 (cancer* or neoplasm* or carcinoma* or malignan* or tumor* or tumour*)).mp. | 578931 |
| 3 | ((breast or mammary) and "invasive ductal carcinoma").mp. | 6391 |
| 4 | Exp breast carcinoma/ | 92812 |
| 5 | Exp breast cancer/ | 451788 |
| 6 | 1 or 2 or 3 or 4 or 5 | 588433 |
| 7 | (microinvasive or microinvasion or T1mi or "micro invasion" or "micro invasive").mp. | 4012 |
| 8 | Exp intraductal carcinoma/ | 15799 |
| 9 | Exp carcinoma in situ/ | 49110 |
| 10 | ("ductal carcinoma in situ" or DCIS or Tis or "intraductal carcinoma").mp. | 25936 |
| 11 | 8 or 9 or 10 | 57069 |
| 12 | 6 and 7 and 11 | 707 |

Cochrane Library

|  | **Formula** | **Results (n)** |
| --- | --- | --- |
| 1 | MeSH descriptor: [Breast Neoplasms] | 12708 |
| 2 | (breast OR mammary) NEAR/2 (cancer* OR neoplasm* OR carcinoma* OR malignan* OR "tumor*" OR "tumour*") | 36986 |
| 3 | (breast OR mammary) AND "invasive ductal carcinoma" | 126 |
| 4 | MeSH descriptor: [Carcinoma, Ductal, Breast] | 352 |
| 5 | #1 OR #2 OR #3 OR #4 | 36996 |
| 6 | microinvasive OR microinvasion OR T1mi OR “micro invasion” OR “micro invasive” | 120 |
| 7 | MeSH descriptor: [Carcinoma, Intraductal, Noninfiltrating] | 179 |
| 8 | “ductal carcinoma in situ” OR DCIS OR Tis OR “intraductal carcinoma” | 1361 |
| 9 | #7 OR #8 | 1439 |
| 10 | #5 AND #6 AND #9 | 15 |

**Supplementary Table S4.**

Differences of clinicopathological characteristics between DCIS-Mi and DCIS group on the 26 studies

| Author | Histological type | Total cases: n (%) | Median age (range) | Menopausal status: n (%) | | | Tumor size: n (%) | | | | Axillary LN status: n (%) | | |
| --- | --- | --- | --- | --- | --- | --- | --- | --- | --- | --- | --- | --- | --- |
|  |  |  |  | pre | post | unknown | ≤2cm | 2cm< and ≤5cm | >5cm | Unknown | Negative | Positive | Unknown |
| Kim, A et al. (2016)  ^#1^ | DCIS-Mi | 27 | 48 (27–76) | NA | | | NA | | | | NA | | |
|  | DCIS | 177 |  |  |  |  |  |  |  |  |  |  |  |
| Beguinot M, et al. (2018) ^#2^ | DCIS-Mi | 35 | 54 (38–78) ^*1^ | NA | | | NA | 10 (28.6) | | NA | NA | | |
|  | DCIS | 96 | 56 (36–84) ^*1^ |  |  |  |  | 21 (21.9) | |  |  |  |  |
| Fang Y, et al. (2016) | DCIS-Mi | 84 | Age<50: n=27 (32.1%), Age≥50: n=57 (67.9%) | 32 (38.1) | 52 (61.9) | 0 (0) | 44 (52.4) | 34 (40.4) | 3 (3.6) | 3 (3.6) | 73 (86.9) | 6 (7.1) | 5 (6.0) |
|  | DCIS | 359 | Age<50: n=157 (43.7%), Age≥50: n=202 (56.3%) | 178 (49.6) | 181 (50.4) | 0 (0) | 207 (57.7) | 116 (32.3) | 12 (3.3) | 24 (6.7) | 293 (81.6) | 4 (1.1) | 62 (17.3) |
| Toss A, et al. (2016)  ^#3^ | DCIS-Mi | 58 | 59.1 (12.7) ^*2^ | NA | | | NA | | | | NA | | |
|  | DCIS | 807 | 58.9 (11.6) ^*2^ |  |  |  |  |  |  |  |  |  |  |
| Wang L, et al. (2015) | DCIS-Mi | 131 | Age<40: n=25 (19.1%), Age≥40: n=106 (80.9%) | 81 (61.8) | 50 (38.2) | 0 (0) | 48 (36.6) | 75 (57.3) | 8 (6.1) | 0 (0) | 121 (92.4) | 10 (7.6) | 0 (0) |
|  | DCIS | 451 | Age<40: n=69 (15.3%), Age≥40: n=382 (84.7%) | 259 (57.4) | 192 (42.6) | 0 (0) | 224 (49.7) | 210 (46.5) | 17 (3.8) | 0 (0) | 447 (99.1) | 4 (0.9) | 0 (0) |
| Zheng J, et al. (2020) | DCIS-Mi | 92 | Age≥50: n=43 (46.7%), Age<50: n=49 (53.3%) | 52 (56.5) | 40 (43.5) | 0 (0) | 67 (72.8) | 20 (21.7) | 5 (5.5) | 0 (0) | 85 (92.4) | 4 (4.3) | 3 (3.3) |
|  | DCIS | 308 | Age≥50: n=143 (46.4%), Age<50: n=165 (53.6%) | 192 (62.3) | 116 (37.7) | 0 (0) | 188 (61.0) | 77 (25.0) | 8 (2.6) | 35 (11.4) | 283 (91.9) | 0 (0) | 25 (8.1) |
| Yu KD, et al. (2011) | DCIS-Mi | 67 | 50.1±10.3 ^*2^ | 36 (53.7) | 31 (46.3) | 0 (0) | <1.5cm: 21 (31.3)  1.5-4cm: 36 (53.7)  ≥4cm: 3 (4.5) | | | 7 (10.4) | 55 (82.1) | 3 (4.5) | 9 (13.4) |
|  | DCIS | 271 | 52±11.8 ^*2^ | 125 (46.1) | 146 (53.9) | 0 (0) | <1.5cm: 120 (44.3) 1.5-4cm: 93 (34.3) ≥4cm: 9 (3.3) | | | 49 (18.1) | 178 (65.7) | 1 (0.3) | 92 (34.0) |
| Zhang W, et al. (2012) ^#4^ | DCIS-Mi | 72 | 49.3 ± 1.01 ^*2^ | 44 (61.1) | 28 (38.9) | 0 (0) | 32 (44.4) ^*3^ | 40 (55.6) ^*4^ | | 0 (0) | 57 (79.2) | 15 (20.8) | 0 (0) |
|  | DCIS | 73 | 48.9 ± 1.13 ^*2^ | 52 (71.2) | 21 (28.8) | 0 (0) | 38 (52.1) ^*3^ | 35 (47.9) ^*4^ | | 0 (0) | 68 (93.2) | 5 (6.8) | 0 (0) |
| Seo YY, et al. (2017)  ^#5^ | DCIS-Mi | 19 | 48 ± 8 ^*2^ | NA | | | 3.5±2.3 ^*2^ | | | | NA | | |
|  | DCIS | 68 | 51 ± 9 ^*2^ |  |  |  | 2.3± 1.7 ^*2^ | | | |  |  |  |
| Parikh RR, et al. (2012) | DCIS-Mi | 72 | Age<50: n=22 (30.6%), Age≥50: n=50 (69.4%) | NA | | | NA | | | | 45 (62.5) | 1 (1.4) | 26 (36.1) |
|  | DCIS | 321 | Age<50: n=113 (35.2%), Age≥50: n=208 (64.8%) |  |  |  |  |  |  |  | 70 (21.8) | 0 (0) | 251 (78.2) |
| Wan ZB, et al. (2018)  ^#6^ | DCIS-Mi | 55 | 46 (24–69) | NA | | | NA | | | | 20 (36.4) | 4 (7.2) | 31 (56.4) |
|  | DCIS | 164 | 46 (21–75) |  |  |  |  |  |  |  | 47 (28.7) | 1 (0.6) | 116 (70.7) |
| González LO, et al. (2010)  ^#7^ | DCIS-Mi | 12 | Age<55: n=6 (50.0%), Age>55: n=6 (50.0%) | 3 (25.0) | 9 (75.0) | 0 (0) | NA | | | | NA | | |
|  | DCIS | 50 | Age<55: n=29 (58.0%), Age>55: n=21 (42.0%) | 18 (36.0) | 32 (64.0) | 0 (0) |  |  |  |  |  |  |  |
| Ozkan-Gurdal S, et al. (2014) ^#8^ | DCIS-Mi | 37 | 51 (22-75) | 14 (37.8) | 23 (62.2) | 0 (0) | NA | 30 (81.1) ^*6^ | | NA | 32 (86.5) | 2 (5.4) | 3 (8.1) |
|  | DCIS | 88 | 50 (29-77) | 35 (39.8) | 53 (60.2) | 0 (0) |  | 50 (56.8) ^*6^ | |  | 48 (54.6) | 1 (1.1) | 39 (44.3) |
| Okumura Y, et al. (2008) ^#9^ | DCIS-Mi | 28 | 50 (32-71) | NA | | | NA | | | | NA | | |
|  | DCIS | 52 | 56 (36-86) |  |  |  |  |  |  |  |  |  |  |
| Wang H, et al. (2019) ^#10^ | DCIS-Mi | 53 | 46 ± 8 ^*2^ | 37 (69.8) | 16 (30.2) | 0 (0) | 3.4±1.5 cm ^*2^ | | | | NA | | |
|  | DCIS | 94 | 48± 11 ^*2^ | 63 (67.0) | 31 (33.0) | 0 (0) | 2.1±1.5 cm ^*2^ | | | |  |  |  |
| Mamtani A, et al. (2019) | DCIS-Mi | 421 | Age<50: n=208 (49.4%), Age ≥50: n=213 (50.6%) | NA | | | NA | | | | NA | | |
|  | DCIS | 2700 | Age<50: n=1381 (51.1%), Age≥50: n=1319 (48.9%) |  |  |  |  |  |  |  |  |  |  |
| Pu T, et al. (2018) | DCIS-Mi | 242 | Age<50: n=143 (59.1%), Age≥50: n=99 (40.9%) | NA | | | 107 (44.2) | 111 (45.9) | 24 (9.9) | 0 (0) | 222 (91.7) | 20 (8.3) | 0 (0) |
|  | DCIS | 280 | Age<50: n=168 (60.0%), Age≥50: n=112 (40.0%) |  |  |  | 146 (52.1) | 122 (43.6) | 12 (4.3) | 0 (0) | 280 (100) | 0 (0) | 0 (0) |
| Costarelli L, et al. (2019) ^#11^ | DCIS-Mi | 233 | 56.4±13 ^*1^ | NA | | | 2.8 ± 1.7 cm^*1^ | | | | 178 (76.4) | 25 (10.7) | 30 (12.9) |
|  | DCIS | 2107 | 57.8± 12.2 ^*1^ |  |  |  | 1.9 ± 1.2 cm^*1^ | | | | 2107 (100) | 0 (0) | 0 (0) |
| Kim M, et al. (2018) | DCIS-Mi | 136 | Age<50: n=69 (50.7%), Age≥50: n=67 (49.3%) | NA | | | <3.2 cm: 52 (38.2) ≥3.2 cm: 84 (61.8) | | | | 106 (78.0) | 4 (2.9) | 26 (19.1) |
|  | DCIS | 477 | Age<50: n=261 (54.7%), Age≥50: n=216 (45.3%) |  |  |  | <3.2cm: 310 (65.0) ≥3.2cm: 167 (35.0) | | | | 190 (39.8) | 0 (0) | 287 (60.2) |
| Hahn SY, et al. (2013) ^#12^ | DCIS-Mi | 37 | 48.1 ± 8.1 | NA | | | 4.3±2.8 cm | | | | NA | 2 (5.4) | NA |
|  | DCIS | 44 | 48.5 ± 9.5 |  |  |  | 3.2±2.3 cm | | | |  | 0 (0) |  |
| Rakovitch E, et al. (2019) | DCIS-Mi | 267 | 57 (IQR: 50–68) | NA | | | <1.0cm: 25 (9.4) ≥1.0cm: 143 (53.5) | | | 99 (37.1) | NA | | |
|  | DCIS | 2721 | 58 (IQR: 50–68) |  |  |  | <1.0cm: 509 (18.7) ≥1.0cm: 913 (33.6) | | | 1299 (47.7) |  |  |  |
| Meretoja TJ, et al. (2012) ^#13^ | DCIS-Mi | 34 | 56 (39–84) | NA | | | 2.3cm (range: 0.4–10) ^*7^ | | | | 27 (79.4) | 7 (20.6) | 0 (0) |
|  | DCIS | 246 | 58 (37–92) |  |  |  | 2.8cm (range: 0.1–13) ^*7^ | | | | 232 (94.3) | 14 (5.7) | 0 (0) |
| Mori M, et al. (2013) ^#14^ | DCIS-Mi | 32 | 55 (32–80) | NA | | | 3.5cm (range: 0.7–11) ^*7^ | | | | 26 (81.3) | 2 (6.2) | 4 (12.5) |
|  | DCIS | 392 | 47 (22–86) |  |  |  | 3.2cm (range: 0.2–14.5) ^*7^ | | | | 293 (74.7) | 9 (2.3) | 90 (23.0) |
| Sue GR, et al. (2013) | DCIS-Mi | 51 | 54.9 | NA | | | 1.5cm^*8^ | | | | NA | | |
|  | DCIS | 154 | 56.7 |  |  |  | 0.9 cm^*8^ | | | |  |  |  |
| Bertozzi S, et al. (2019) | DCIS-Mi | 84 | 58.8 ± 11.7 ^*2^ | NA | 64 (76.2) | NA | NA | | | | 76 (90.5) | 8 (9.5) | 0 (0) |
|  | DCIS | 543 | 58.8 ± 11.4 ^*2^ |  | 422 (77.7) |  |  |  |  |  | 529 (97.4) | 14 (2.6) | 0 (0) |
| Yao JJ, et al. (2015) ^#15^ | DCIS-Mi | 58 | 55 ± 13 ^*2^ | 31 (53.4) | 27 (46.6) | 0 (0) | 16 (27.6) | 39 (67.2) | 3 (5.2) | 0 (0) | 55 (94.8) | 3 (5.2) | 0 (0) |
|  | DCIS | 160 | 53 ± 10 ^*2^ | 91 (56.9) | 69 (43.1) | 0 (0) | 76 (47.5) | 84 (52.5) | 0 (0) | 0 (0) | 160 (100) | 0 (0) | 0 (0) |

| Author | Histological type | Comedo necrosis: n (%) | Definition of tumor grade | Nuclear grade: n (%) | | | | Number of microinvasive foci: n (%) | | | | Primary surgery: n (%) | | | Margin status: n (%) | | |
| --- | --- | --- | --- | --- | --- | --- | --- | --- | --- | --- | --- | --- | --- | --- | --- | --- | --- |
|  |  |  |  | 1 | 2 | 3 | Unknown | 1 | ≥2 | Unknown | | BCS | Bt | Unknown | Positive | Negative | Unknown |
| Kim, A et al. (2016) | DCIS-Mi | 26 (96.3) ^*9^ | NA | 0 (0) | 8 (29.6) | 19 (70.4) | 0 (0) | NA | | | | NA | | | NA | | |
|  | DCIS | 118 (66.7) ^*9^ |  | 27 (15.2) | 89 (50.3) | 61 (34.5) | 0 (0) |  |  |  |  |  |  |  |  |  |  |
| Beguinot M, et al. (2018) | DCIS-Mi | 14 (40.0) | NA | 2 (5.7) | 12 (34.3) | 21 (60.0) | 0 (0) | NA | | | | 21 (60.0) | 14 (40.0) | 0 (0) | NA | | |
|  | DCIS | 26 (27.1) |  | 14 (14.6) | 45 (46.9) | 37 (38.5) | 0 (0) |  |  |  |  | 68 (70.8) | 28 (29.2) | 0 (0) |  |  |  |
| Fang Y, et al. (2016) | DCIS-Mi | NA | NA | NA | | | | 51 (60.7) | 33 (39.3) | | 0 (0) | 25 (29.8) | 59 (70.2) | 0 (0) | NA | | |
|  | DCIS |  |  |  |  |  |  | Not related | | | | 86 (24.0) | 273 (76.0) | 0 (0) |  |  |  |
| Toss A, et al. (2016) | DCIS-Mi | NA | NA | NA | | | | NA | | | | 33 (56.9) | 24 (41.4) | 1 (1.7) | NA | | |
|  | DCIS |  |  |  |  |  |  |  |  |  |  | 600 (74.3) | 203 (25.2) | 4 (0.5) |  |  |  |
| Wang L, et al. (2015) | DCIS-Mi | NA | NA | 41 (31.3) | 64 (48.9) | 26 (19.8) | 0 (0) | NA | | | | NA | | | NA | | |
|  | DCIS |  |  | 174 (38.6) | 195 (43.2) | 82 (18.2) | 0 (0) |  |  |  |  |  |  |  |  |  |  |
| Zheng J, et al. (2020) | DCIS-Mi | NA | NA | 21 (22.8) | | 57 (62.0) | 14 (15.2) | NA | | | | 5 (5.4) | 87 (94.6) | 0 (0) | NA | | |
|  | DCIS |  |  | 170 (55.2) | | 116 (37.7) | 22 (7.1) |  |  |  |  | 53 (17.2) | 255 (82.8) | 0 (0) |  |  |  |
| Yu KD, et al. (2011) | DCIS-Mi | NA | NA | 7 (10.4) | 17 (25.4) | 11 (16.4) | 32 (47.8) | NA | | | | NA | | | NA | | |
|  | DCIS |  |  | 48 (17.7) | 75 (27.7) | 24 (8.8) | 124 (45.8) |  |  |  |  |  |  |  |  |  |  |
| Zhang W, et al. (2012) | DCIS-Mi | NA | NA | NA | | | | NA | | | | NA | | | NA | | |
|  | DCIS |  |  |  |  |  |  |  |  |  |  |  |  |  |  |  |  |
| Seo YY, et al. (2017) | DCIS-Mi | NA | Nottingham grading system | 0 (0) | 3 (15.8) | 16 (84.2) | 0 (0) | NA | | | | 8 (42.1) | 11 (57.9) | 0 (0) | NA | | |
|  | DCIS |  |  | 15 (22.0) | 21 (30.9) | 32 (47.1) | 0 (0) |  |  |  |  | 40 (58.8) | 28 (41.2) | 0 (0) |  |  |  |
| Parikh RR, et al. (2012) | DCIS-Mi | 26 (36.1) | NA | NA | | | | NA | | | | NA | | | 13 (18.1) ^*13^ | 59 (81.9) | 0 (0) |
|  | DCIS | 76 (23.7) |  |  |  |  |  |  |  |  |  |  |  |  | 85 (26.5) ^*13^ | 236 (73.5) | 0 (0) |
| Wan ZB, et al. (2018) | DCIS-Mi | NA | NA | 4 (7.3) ^*11^ | 38 (69.1) ^*11^ | 13 (23.6) ^*11^ | 0 (0) | NA | | | | NA | | | NA | | |
|  | DCIS |  |  | 65 (39.6) ^*11^ | 89 (54.3) ^e*11^ | 10 (6.1) ^*11^ | 0 (0) |  |  |  |  |  |  |  |  |  |  |
| González LO, et al. (2010) | DCIS-Mi | 2 (16.6) | Van Nuys classification | 7 (58.4) | 1 (8.3) | 4 (33.3) | 0 (0) | NA | | | | NA | | | NA | | |
|  | DCIS | 4 (8.0) |  | 21 (42.0) | 6 (12.0) | 23 (46.0) | 0 (0) |  |  |  |  |  |  |  |  |  |  |
| Ozkan-Gurdal S, et al. (2014) | DCIS-Mi | 23 (62.2) | NA | NA | NA | 25 (67.6) ^*10^ | NA | NA | | | | 14 (37.8) | 23 (62.2) | 0 (0) | NA | | |
|  | DCIS | 27 (30.7) |  |  |  | 34 (38.6) ^*10^ |  |  |  |  |  | 37 (42.0) | 51 (58.0) | 0 (0) |  |  |  |
| Okumura Y, et al. (2008) | DCIS-Mi | 10 (35.7) ^*9^ | Van Nuys classification | 7 (25.0) | 18 (64.3) | 3 (10.7) | 0 (0) | NA | | | | NA | | | NA | | |
|  | DCIS | 8 (15.4) ^*9^ |  | 21 (40.4) | 29 (55.8) | 2 (3.8) | 0 (0) |  |  |  |  |  |  |  |  |  |  |
| Wang H, et al. (2019) | DCIS-Mi | 17 (32.1) | NA | 11 (20.8) | | 42 (79.2) | 0 (0) | NA | | | | NA | | | NA | | |
|  | DCIS | 3 (3.2) |  | 68 (72.3) | | 26 (27.7) | 0 (0) |  |  |  |  |  |  |  |  |  |  |
| Mamtani A, et al. (2019) | DCIS-Mi | NA | NA | NA | | | | NA | | | | NA | | | NA | | |
|  | DCIS |  |  |  |  |  |  |  |  |  |  |  |  |  |  |  |  |
| Pu T, et al. (2018) | DCIS-Mi | 132 (54.5) ^*9^ | NA | 15 (6.2) | 64 (26.4) | 163 (67.4) | 0 (0) | NA | | | | 26 (10.7) | 216 (89.3) | 0 (0) | NA | | |
|  | DCIS | 143 (51.1) ^*9^ |  | 21 (7.5) | 126 (45.0) | 133 (47.5) | 0 (0) |  |  |  |  | 33 (11.8) | 247 (88.2) | 0 (0) |  |  |  |
| Costarelli L, et al. (2019) | DCIS-Mi | NA | NA | 84 (36.1) ^*11^ | | 86 (36.9) | 63 (27.0) | NA | | | | 132 (56.7) | NA | 1 (0.4) | NA | | |
|  | DCIS |  |  | NA | | | |  |  |  |  | 1526 (72.4) |  | 50 (2.4) |  |  |  |
| Kim M, et al. (2018) | DCIS-Mi | 85 (62.5) | NA | 32 (23.5) | | 104 (76.5) | 0 (0) | NA | | | | 55 (40.4) | 81 (59.6) | 0 (0) | NA | | |
|  | DCIS | 102 (21.4) |  | 300 (62.9) | | 177 (37.1) | 0 (0) |  |  |  |  | 316 (66.2) | 161 (33.8) | 0 (0) |  |  |  |
| Hahn SY, et al. (2013) | DCIS-Mi | NA | Van Nuys classification | 11 (29.7) | | 26 (70.3) | 0 (0) | NA | | | | 28 (75.7) | 9 (24.3) | 0 (0) | NA | | |
|  | DCIS |  |  | 29 (65.9) | | 15 (34.1) | 0 (0) |  |  |  |  | 26 (59.1) | 18 (40.9) | 0 (0) |  |  |  |
| Rakovitch E, et al. (2019) | DCIS-Mi | 220 (82.4) ^*9^ | NA | Low/intermediate/unknown: 91 (34.1), high: 176 (65.9) | | | | 156 (58.4) | 111 (41.6) | 0 (0) | | 267 (100) | 0 (0) | 0 (0) | Positive/unknown: 47 (17.6), Negative: 220 (82.4) ^*5^ | | |
|  | DCIS | 1662 (61.1) ^*9^ |  | Low/intermediate/unknown: 1709 (62.8), high: 1012 (37.2) | | | | Not related | | | | 2721 (100) | 0 (0) | 0 (0) | Positive/unknown: 622 (22.9) Negative: 2099 (77.1) ^*5^ | | |
| Meretoja TJ, et al. (2012) | DCIS-Mi | 19 (55.9) | Van Nuys classification | 7 (20.6) ^*12^ | 5 (14.7) ^*12^ | 22 (64.7) ^*12^ | 0 (0) | NA | | | | 15 (44.1) | 19 (55.9) | 0 (0) | NA | | |
|  | DCIS | 132 (53.7) |  | 30 (12.2) ^*12^ | 59 (24.0) ^*12^ | 157 (63.8) ^*12^ | 0 (0) |  |  |  |  | 86 (35.0) | 160 (65.0) | 0 (0) |  |  |  |
| Mori M, et al. (2013) | DCIS-Mi | 22 (68.8) | NA | 8 (25.0) | 8 (25.0) | 16 (50.0) | 0 (0) | NA | | | | NA | | | NA | | |
|  | DCIS | 54 (13.8) |  | 297 (75.8) | 62 (15.8) | 33 (8.4) | 0 (0) |  |  |  |  |  |  |  |  |  |  |
| Sue GR, et al. (2013) | DCIS-Mi | 40 (78.4) | NA | 1 (2.0) ^*11^ | 21 (41.2) ^*11^ | 27 (52.9) ^*11^ | 2 (3.9) | NA | | | | NA | | | NA | | |
|  | DCIS | 59 (38.3) |  | 15 (9.7) ^*11^ | 70 (45.5) ^*11^ | 38 (24.7) ^*11^ | 31 (20.1) |  |  |  |  |  |  |  |  |  |  |
| Bertozzi S, et al. (2019) | DCIS-Mi | 37(44.0) | AJCC/UICC, 2009 and the recommendations of AFIP (DCIS) | 5 (5.9) ^*11^ | 45 (53.6) ^*11^ | 34 (40.5) ^*11^ | 0 (0) | NA | | | | 30 (35.7) | 54 (64.3) | 0 (0) | NA | | |
|  | DCIS | 149 (27.4) |  | 99 (18.2) ^*11^ | 265 (48.8) ^*11^ | 179 (33.0) ^*11^ | 0 (0) |  |  |  |  | 319 (58.7) | 224 (41.3) | 0 (0) |  |  |  |
| Yao JJ, et al. (2015) | DCIS-Mi | NA | Van Nuys classification | 4 (6.9) | 17 (29.3) | 37 (63.8) | 0 (0) | NA | | | | NA | | | NA | | |
|  | DCIS |  |  | 43 (26.9) | 54 (33.8) | 63 (39.3) | 0 (0) |  |  |  |  |  |  |  |  |  |  |

| Author | Histological type | Ki67 labeling index: n (%) | | | Definition of ER/PR positivity | Definition of HER2 positivity | ER status: n (%) | | | PR status: n (%) | | | HER2 status: n (%) | | |
| --- | --- | --- | --- | --- | --- | --- | --- | --- | --- | --- | --- | --- | --- | --- | --- |
|  |  | <20% | ≥20% | Unknown |  |  | Positive | Negative | Unknown | Positive | Negative | Unknown | Positive | Negative | Unknown |
| Kim, A et al. (2016) | DCIS-Mi | NA | | | ≥ 1% | 3+ or gene amplification as identified by SISH | NA | | | NA | | | NA | | |
|  | DCIS |  |  |  |  |  |  |  |  |  |  |  |  |  |  |
| Beguinot M, et al. (2018) | DCIS-Mi | 5.6± 6.4 | | | According to Allred score ^#16^ | ASCO/CAP criteria ^#17^ | NA | | | NA | | | NA | | |
|  | DCIS | 4.8± 7.3 | | |  |  |  |  |  |  |  |  |  |  |  |
| Fang Y, et al. (2016) | DCIS-Mi | 18.25 ± 14.65^*2^ | | | ≥ 1% | HER2 3+ by IHC or positive on FISH | 42 (50.0) | 42 (50.0) | 0 (0) | 32 (38.1) | 52 (61.9) | 0 (0) | 36 (42.9) | 48 (57.1) | 0 (0) |
|  | DCIS | 14.29 ± 14.26^*2^ | | |  |  | 249 (69.4) | 107 (29.8) | 3 (0.8) | 208 (58.0) | 148 (41.2) | 3 (0.8) | 104 (28.9) | 249 (69.4) | 6 (1.7) |
| Toss A, et al. (2016) | DCIS-Mi | NA | | | ≥10% positive, 1–9% borderline | More than 30% with IHC or amplified with FISH test, 10-29% borderline | 35 (60.3) | 11 (19.0) | 12 (20.7) | 29 (50.0) | 17 (29.3) | 12 (20.7) | 10 (17.2) | 9 (15.5) | 39 (67.3) ^*22^ |
|  | DCIS |  |  |  |  |  | 524 (64.9) | 86 (10.7) | 197 (24.4) | 464 (57.5) | 142 (17.6) | 201 (24.9) | 77 (9.5) | 63 (7.8) | 667 (82.7) ^*22^ |
| Wang L, et al. (2015) | DCIS-Mi | 40 (30.5) | 91 (69.5) | 0 (0) | More than 1 % | Strong whole membrane staining in >10 % of the tumor cells | 82 (62.6) | 49 (37.4) | 0 (0) | 76 (58.0) | 55 (42.0) | 0 (0) | 68 (51.9) | 63 (48.1) | 0 (0) |
|  | DCIS | 160 (35.5) | 291 (64.5) | 0 (0) |  |  | 297 (65.9) | 154 (34.1) | 0 (0) | 276 (61.2) | 175 (38.8) | 0 (0) | 263 (58.3) | 188 (41.7) | 0 (0) |
| Zheng J, et al. (2020) | DCIS-Mi | 56 (60.9) | 36 (39.1) | 0 (0) | ≥1% | IHC 3+, 2+ (tested by FISH) | 71 (77.2) | 21 (22.8) | 0 (0) | 64 (69.6) | 28 (30.4) | 0 (0) | 42 (45.6) | 26 (28.3) | 24 (26.1) |
|  | DCIS | 211 (68.5) | 97 (31.5) | 0 (0) |  |  | 251 (81.5) | 57 (18.5) | 0 (0) | 240 (77.9) | 68 (22.1) | 0 (0) | 96 (31.2) | 116 (37.6) | 96 (31.2) |
| Yu KD, et al. (2011) | DCIS-Mi | NA | | | Scores of 1–12 (Proportion score and intensity score.) | Strong membranous staining with scores of 9–12 (Score 3+) | 31 (46.3) | 30 (44.8) | 6 (8.9) | 27 (40.3) | 34 (50.7) | 6 (9.0) | 23 (34.3) | 38 (56.7) | 6 (9.0) |
|  | DCIS |  |  |  |  |  | 140 (51.7) | 68 (25.1) | 63 (23.2) | 109 (40.2) | 98 (36.2) | 64 (23.6) | 69 (25.5) | 136 (50.2) | 66 (24.3) |
| Zhang W, et al. (2012) | DCIS-Mi | NA | | | A score of 1: no stain, 1: ≤25% of cells positive, 2: 25-50%, 3: 50-75%, 4: >75% | | 42 (58.3) | 30 (41.7) | 0 (0) | 37 (51.4) | 35 (48.6) | 0 (0) | 48 (66.7) | 24 (33.3) | 0 (0) |
|  | DCIS |  |  |  |  |  | 44 (60.3) | 29 (39.7) | 0 (0) | 41 (56.2) | 32 (43.8) | 0 (0) | 47 (64.4) | 26 (35.6) | 0 (0) |
| Seo Y, et al. (2017) | DCIS-Mi | 22.0 ± 21^*5^ | | | More than 10% | 3+ by IHC, 2+: positive on FISH | 2 (10.5) | 17 (89.5) | 0 (0) | 4 (21.1) | 15 (78.9) | 0 (0) | 14 (73.7) | 5 (26.3) | 0 (0) |
|  | DCIS | 9.5 ± 7.8 ^*5^ | | |  |  | 47 (69.1) | 21 (30.9) | 0 (0) | 43 (63.2) | 25 (36.8) | 0 (0) | 31 (45.6) | 37 (54.4) | 0 (0) |
| Parikh RR, et al. (2012) | DCIS-Mi | NA | | | NA | NA | 7 (9.7) | 5 (7.0) | 60 (83.3) | 4 (5.6) | 5 (6.9) | 63 (87.5) | NA | | |
|  | DCIS |  |  |  |  |  | 3 (0.9) | 7 (2.2) | 311 (96.9) | 3 (0.9) | 6 (1.9) | 312 (97.2) |  |  |  |
| Wan ZB, et al. (2018) | DCIS-Mi | 23 (41.8) ^*14^ | 32 (58.2) ^*15^ | 0 (0) | ≥ 1% | 3+ by IHC, 2+: positive on FISH | 25 (45.5) | 30 (54.5) | 0 (0) | 13 (23.6) | 42 (76.4) | 0 (0) | 31 (56.4) | 24 (43.6) | 0 (0) |
|  | DCIS | 83 (50.6) ^*14^ | 81 (49.4) ^*15^ | 0 (0) |  |  | 118 (72.0) | 46 (28.0) | 0 (0) | 88 (53.7) | 76 (46.3) | 0 (0) | 60 (36.6) | 104 (63.4) | 0 (0) |
| González LO, et al. (2010) | DCIS-Mi | NA | | | Allred score: 0–3, negative; 4–9 positive) | NA | 8 (66.7) | 4 (33.3) | 0 (0) | 7 (58.3) | 5 (41.6) | 0 (0) | NA | | |
|  | DCIS |  |  |  |  |  | 40 (80.0) | 10 (20.0) | 0 (0) | 30 (60.0) | 20 (40.0) | 0 (0) |  |  |  |
| Ozkan-Gurdal S, et al. (2014) | DCIS-Mi | NA | | | ≥ 1% | 3+ on IHC or 2+: positive on FISH | 14 (37.9) | 18 (48.6) | 5 (13.5) | 14 (37.9) | 15 (40.5) | 8 (21.6) | 9 (24.4) | 14 (37.8) | 14 (37.8) |
|  | DCIS |  |  |  |  |  | 48 (54.5) | 13 (14.8) | 27 (30.7) | 41 (46.6) | 21 (23.9) | 26 (29.5) | 3 (3.4) | 13 (14.8) | 72 (81.8) |
| Okumura Y, et al. (2008) | DCIS-Mi | 22.8 ± 2.0 ^*2^ | | | NA | 3+ (strong staining) | 19 (67.9) | 9 (32.1) | 0 (0) | 16 (57.1) | 12 (42.9) | 0 (0) | 6 (21.4) ^*19^ | 22 (78.6) ^*20^ | 0 (0) |
|  | DCIS | 17.9 ± 1.5 ^*2^ | | |  |  | 38 (73.1) | 14 (26.9) | 0 (0) | 37 (71.2) | 15 (28.8) | 0 (0) | 9 (17.3) ^*19^ | 43 (82.7) ^*20^ | 0 (0) |
| Wang H, et al. (2019) | DCIS-Mi | 25 (47.2) ^*14^ | 28 (52.8) ^*15^ | 0 (0) | ≥ 1% | 3+ by IHC | 31 (58.5) | 22 (41.5) | 0 (0) | 16 (30.2) | 37 (69.8) | 0 (0) | 21 (39.6) ^*19^ | 11 (20.8) | 21 (39.6) ^*20^ |
|  | DCIS | 66 (70.2) ^*14^ | 28 (29.8) ^*15^ | 0 (0) |  |  | 86 (91.5) | 8 (8.5) | 0 (0) | 76 (80.9) | 18 (19.1) | 0 (0) | 16 (17.1) ^*19^ | 46 (48.9) | 32 (34.0) ^*20^ |
| Mamtani A, et al. (2019) | DCIS-Mi | NA | | | NA | NA | NA | | | NA | | | NA | | |
|  | DCIS |  |  |  |  |  |  |  |  |  |  |  |  |  |  |
| Pu T, et al. (2018) | DCIS-Mi | 108 (44.6) ^*16^ | 134 (55.4) ^*17^ | 0 (0) | ≥1% | IHC score: 3+ or FISH-positive | 117 (48.3) | 125 (51.7) | 0 (0) | 112 (46.3) | 130 (53.7) | 0 (0) | 109 (45.0) ^*19^ | 84 (34.7) | 49 (20.3) ^*20^ |
|  | DCIS | 109 (38.9) ^*16^ | 171 (61.1) ^*17^ | 0 (0) |  |  | 196 (70.0) | 84 (30.0) | 0 (0) | 172 (61.4) | 108 (38.6) | 0 (0) | 83 (29.6) ^*19^ | 137 (48.9) | 60 (21.4) ^*20^ |
| Costarelli L, et al. (2019) | DCIS-Mi | 69 (29.6) | 80 (34.3) ^*18^ | 84 (36.1) | NA | NA | 131 (56.2) | 73 (31.3) | 29 (12.5) | 102 (43.8) | 101 (43.3) | 30 (12.9) | 49 (21.0) | 71 (30.5) | 113 (48.5) ^*21^ |
|  | DCIS | NA | | |  |  | 920 (43.7) | 222 (10.5) | 965 (45.8) | 799 (37.9) | 331 (15.7) | 977 (46.4) | NA | | |
| Kim M, et al. (2018) | DCIS-Mi | 91 (66.9) | 45 (33.1) | 0 (0) | ≥10% | According to the 2013 ASCO/CAP guideline | 62 (45.6) | 74 (54.4) | 0 (0) | 48 (35.3) | 88 (64.7) | 0 (0) | 78 (57.4) | 58 (42.6) | 0 (0) |
|  | DCIS | 421 (88.3) | 56 (11.7) | 0 (0) |  |  | 394 (82.6) | 83 (17.4) | 0 (0) | 349 (73.2) | 128 (26.8) | 0 (0) | 108 (22.6) | 369 (77.4) | 0 (0) |
| Hahn SY, et al. (2013) | DCIS-Mi | NA | | | NA | NA | NA | | | NA | | | NA | | |
|  | DCIS |  |  |  |  |  |  |  |  |  |  |  |  |  |  |
| Rakovitch E, et al. (2019) | DCIS-Mi | NA | | | NA | NA | NA | | | NA | | | NA | | |
|  | DCIS |  |  |  |  |  |  |  |  |  |  |  |  |  |  |
| Meretoja TJ, et al. (2012) | DCIS-Mi | NA | | | NA | NA | NA | | | NA | | | NA | | |
|  | DCIS |  |  |  |  |  |  |  |  |  |  |  |  |  |  |
| Mori M, et al. (2013) | DCIS-Mi | 35.2 ± 19.2^*5^ | | | 3 or more (Allred score) | 3+, 2+ (unclassified) | NA | | | NA | | | NA | | |
|  | DCIS | 18.8 ± 14.5^*5^ | | |  |  |  |  |  |  |  |  |  |  |  |
| Sue GR, et al. (2013) | DCIS-Mi | NA | | | NA | NA | NA | | | NA | | | NA | | |
|  | DCIS |  |  |  |  |  |  |  |  |  |  |  |  |  |  |
| Bertozzi S, et al. (2019) | DCIS-Mi | NA | 13 (15.5) | 52 (61.9) | ≥1% | 3+ on IHC or 2+: positive on FISH | NA | | | NA | | | NA | | |
|  | DCIS |  | 13 (2.4) | 492 (90.6) |  |  |  |  |  |  |  |  |  |  |  |
| Yao JJ, et al. (2015) | DCIS-Mi | 34 (58.6) ^*14^ | 24 (41.4) ^*15^ | 0 (0) | ≥ 10% | 3+ by IHC, 2+: positive on FISH (HER2/CEP >2.2) | 39 (67.2) | 19 (32.8) | 0 (0) | 36 (62.1) | 22 (37.9) | 0 (0) | 25 (43.1) | 33 (56.9) | 0 (0) |
|  | DCIS | 132 (82.5) ^*14^ | 28 (17.5) ^*15^ | 0 (0) |  |  | 113 (70.6) | 47 (29.4) | 0 (0) | 105 (65.6) | 55 (34.4) | 0 (0) | 44 (27.5) | 116 (72.5) | 0 (0) |

| Author | Histological type | Adjuvant HT: n (%) | | Adjuvant CT: n (%) | | Adjuvant HER2 targeted therapy: n (%) | | Radiotherapy: n (%) | | Description of median follow-up term | Description of survival rates |
| --- | --- | --- | --- | --- | --- | --- | --- | --- | --- | --- | --- |
|  |  | Yes | Unknown | Yes | Unknown | Yes | Unknown | Yes | Unknown |  |  |
| Kim, A et al. (2016) | DCIS-Mi | NA | | NA | | NA | | NA | | 47.0 months (range: 0–57) | The incident rate of LRR: 7.4 % (2/27) |
|  | DCIS |  |  |  |  |  |  |  |  |  | The incident rate of LRR: 1.7 % (3/177) |
| Beguinot M, et al. (2018) | DCIS-Mi | 9 (25.7) | NA | 2 (5.7) | NA | 1 (2.9) | NA | 21 (60.0) | NA | 144 months (range: 115–173) | 5-year DFS rate: 94%, 10-year DFS rate: 91% |
|  | DCIS | 1 (1.0) |  | 0 (0) |  | 0 (0) |  | 68 (70.8) |  |  | 5-year DFS rate: 94%, 10-year DFS rate: 88% |
| Fang Y, et al. (2016) | DCIS-Mi | 41 (48.8) | 0 (0) | 16 (19.0) | 0 (0) | 3 (3.6) | 0 (0) | 29 (34.5) | 0 (0) | 31 months (range: 2–144) | 3-year DFS rate: 89.5%, 3-year OS rate: 100% |
|  | DCIS | 138 (38.4) | 5 (1.4) | 15 (4.2) | 5 (1.4) | 0 (0) | 0 (0) | 55 (15.3) | 2 (0.6) |  | 3-year DFS rate: 97.1%, 3-year OS rate: 99.6% |
| Toss A, et al. (2016) | DCIS-Mi | NA | | 8 (13.8) | 1 (1.7) | NA | | 25 (43.1) | 1 (1.7) | NA | NA |
|  | DCIS |  |  | 14 (1.7) | 56 (6.9) |  |  | 241 (29.9) | 47 (5.8) |  |  |
| Wang L, et al. (2015) | DCIS-Mi | 68 (51.9) | 0 (0) | NA | | NA | | NA | | 69 months | 5-year OS rate: 99.0%, 5-year DFS rate: 95.2% |
|  | DCIS | 273 (60.5) | 0 (0) |  |  |  |  |  |  | 62 months | 5-year OS rate: 99.2%, 5-year DFS rate: 95.9% |
| Zheng J, et al. (2020) | DCIS-Mi | NA | | NA | | NA | | NA | | 25 months | DFS rate: 97.4%, OS rate: 100% |
|  | DCIS |  |  |  |  |  |  |  |  |  | DFS rate: 99.6%, OS rate: 100% |
| Yu KD, et al. (2011) | DCIS-Mi | NA | | NA | | NA | | NA | | 35 months (range: 1–118) | 5-year RFS rate: 93% |
|  | DCIS |  |  |  |  |  |  |  |  |  | 5-year RFS rate: 96% |
| Zhang W, et al. (2012) | DCIS-Mi | NA | | NA | | NA | | NA | | NA | NA |
|  | DCIS |  |  |  |  |  |  |  |  |  |  |
| Seo YY, et al. (2017) | DCIS-Mi | NA | | NA | | NA | | NA | | NA | NA |
|  | DCIS |  |  |  |  |  |  |  |  |  |  |
| Parikh RR, et al. (2012) | DCIS-Mi | 8 (11.1) | 2 (2.8) | NA | | NA | | NA | | 8.94 years | 10-year LRFS rate: 90.7%, 10-year DMFS rate: 97.9%  10-year OS rate: 95.7% |
|  | DCIS | 66 (20.6) | 5 (1.6) |  |  |  |  |  |  |  | 10-year LRFS rate: 89.0%, 10-year DMFS rate: 98.5%  10-year OS rate: 93.2% |
| Wan ZB, et al. (2018) | DCIS-Mi | NA | | NA | | NA | | NA | | NA | NA |
|  | DCIS |  |  |  |  |  |  |  |  |  |  |
| González LO, et al. (2010) | DCIS-Mi | NA | | NA | | NA | | NA | | NA | NA |
|  | DCIS |  |  |  |  |  |  |  |  |  |  |
| Ozkan-Gurdal S, et al. (2014) | DCIS-Mi | NA | | NA | | NA | | NA | | 53 months (range: 12–144) | IBTR rate was 7.7% for patients with BCS alone and 5.3% for patients with BCS and RT. One patient with DCIS who underwent BCS with RT developed distant metastasis. |
|  | DCIS |  |  |  |  |  |  |  |  |  |  |
| Okumura Y, et al. (2008) | DCIS-Mi | NA | | NA | | NA | | NA | | NA | NA |
|  | DCIS |  |  |  |  |  |  |  |  |  |  |
| Wang H, et al. (2019) | DCIS-Mi | NA | | NA | | NA | | NA | | NA | NA |
|  | DCIS |  |  |  |  |  |  |  |  |  |  |
| Mamtani A, et al. (2019) | DCIS-Mi | NA | | NA | | NA | | NA | | 6.4 years (range: 0–23) | The incident rate of LRR: 3.7% (high grade), 1.5% (low/intermediate grade) |
|  | DCIS |  |  |  |  |  |  |  |  |  | The incident rate of LRR: 1.3% (high grade), 0.4% (low/intermediate grade) |
| Pu T, et al. (2018) | DCIS-Mi | NA | | 165 (68.2) | 0 (0) | NA | | 23 (9.5) | NA | 109 months (range: 6–205) | 5-year DFS rate: 96.9%, 5-year OS rate: 99.3% |
|  | DCIS |  |  | NA | |  |  | NA | |  | 5-year DFS rate: 100%, 5-year OS rate: 100% |
| Costarelli L, et al. (2019) | DCIS-Mi | 84 (36.1) | 33 (14.2) | 28 (12.0) | 43 (18.5) | NA | | 101 (43.3) | 112 (48.1) | NA | NA |
|  | DCIS | 388 (18.4) | 645 (30.6) | NA | |  |  | 959 (45.5) | 835 (39.6) |  |  |
| Kim M, et al. (2018) | DCIS-Mi | NA | | NA | | NA | | NA | | 4.0 years (range: 0.1–12.4) | IBTR rate: 3.5% |
|  | DCIS |  |  |  |  |  |  |  |  |  | IBTR rate: 8.8% |
| Hahn SY, et al. (2013) | DCIS-Mi | NA | | NA | | NA | | NA | | NA | NA |
|  | DCIS |  |  |  |  |  |  |  |  |  |  |
| Rakovitch E, et al. (2019) | DCIS-Mi | 19 (7.1) | NA | NA | | NA | | 154 (57.7) | NA | 13 years (range: 11–16) | 15-year LRFS rate: 67.7% or 62.3% (BCS alone), 81.8% or 70.3% (BCS+RT no boost), 91.3% or 90.0% (BCS + RT with boost) |
|  | DCIS | 145 (5.3) |  |  |  |  |  | 1376 (50.6) |  |  | 15-year LRFS rate: 73.7% (BCS alone), 80.3% (BCS+RT no boost), 82.3% (BCS+RT with boost) |
| Meretoja TJ, et al. (2012) | DCIS-Mi | 4 (11.8) | NA | 4 (11.8) | NA | NA | | 14 (41.2) | NA | 50 months (range: 7–123) | Ipsilateral breast recurrence: DCIS (1 case)  No contralateral breast  No axillary LN recurrence |
|  | DCIS | 5 (2.0) |  | 1 (0.4) |  |  |  | 67 (27.2) |  |  | Ipsilateral breast recurrence: invasive carcinoma (2 cases), DCIS (2 cases)  Contralateral breast: invasive carcinoma (3 cases), DCIS (3 cases)  Axillary LN recurrence: 1 case |
| Mori M, et al. (2013) | DCIS-Mi | NA | | NA | | NA | | NA | | NA | NA |
|  | DCIS |  |  |  |  |  |  |  |  |  |  |
| Sue GR, et al. (2013) | DCIS-Mi | NA | | NA | | NA | | NA | | 8.5 years | NA |
|  | DCIS |  |  |  |  |  |  |  |  |  |  |
| Bertozzi S, et al. (2019) | DCIS-Mi | 51 (60.7) | 2 (2.4) | 11 (13.1) | 2 (2.4) | NA | | 29 (34.5) | 2 (2.4) | NA | 5-year OS rate: 98.8%, 5-year local recurrence rate: 7.9%, 5-year distant recurrence rate: 4% |
|  | DCIS | 253 (46.6) | 55 (10.1) | 25 (4.6) | 57 (10.5) |  |  | 255 (47.0) | 52 (9.6) |  | 5-year OS rate: 99.8%, 5-year local recurrence rate: 2.5%, 5-year distant recurrence rate: 0% |
| Yao JJ, et al. (2015) | DCIS-Mi | NA | | NA | | NA | | NA | | NA | NA |
|  | DCIS |  |  |  |  |  |  |  |  |  |  |

Abbreviations: BCS, breast-conserving surgery; Bt, breast mastectomy; CT, chemotherapy; DCIS-Mi, ductal carcinoma *in situ* with microinvasion; DCIS, ductal carcinoma *in situ*; DFS, disease-free survival; DMFS, distant metastasis-free survival; ER, estrogen receptor; FISH, fluorescence *in situ* hybridization; HER2, human epidermal growth factor receptor 2; HT, hormone therapy; IBTR, ipsilateral breast tumor recurrence; IHC, immunohistochemical; IQR, interquartile range; LN, lymph node; LRFS, loco-regional recurrence-free survival; LRR, loco-regional recurrence; NA, not available; OS, overall survival; PgR, progesterone receptor; RFS, recurrence-free survival; RT, radiotherapy; SD, standard deviation; SISH, silver *in situ* hybridization.

^*1^ Mean (range)

^*2^ Mean ± SD

^*3^ < 2cm

^*4^ ≥ 2cm

^*5^ Details of the definition were not available.

^*6^ > 1.5cm

^*7^ Median (range)

^*8^ Median

^*9^ Described as “necrosis”.

^*10^ Described as “high nuclear grade”

^*11^ Described as “grade”. The details of whether nuclear grade or histological grade were not available.

^*12^ Histological grade

^*13^ ≤2mm

^*14^ ≤20%

^*15^ > 20%

^*16^ ≤14%

^*17^ >14%

^*18^ >15%

^*19^ The cases with IHC 3+ were only included.

^*20^ The cases with IHC 2+ were included.

^*21^ The cases with equivocal were included.

^*22^ The cases with borderline were included.

#1 Kim A, Heo SH, Kim YA et al (2016) An Examination of the Local Cellular Immune Response to Examples of Both Ductal Carcinoma In Situ (DCIS) of the Breast and DCIS With Microinvasion, With Emphasis on Tertiary Lymphoid Structures and Tumor Infiltrating Lymphoctytes. Am J Clin Pathol 146:137-44

#2 Beguinot M, Dauplat MM, Kwiatkowski F et al (2018) Analysis of tumour-infiltrating lymphocytes reveals two new biologically different subgroups of breast ductal carcinoma in situ. BMC Cancer 18:129

#3 Toss A, Palazzo J, Berger A et al (2016) Clinical-pathological features and treatment modalities associated with recurrence in DCIS and micro-invasive carcinoma: Who to treat more and who to treat less. Breast 29:223-30

#4 Zhang W, Gao EL, Zhou YL et al (2012) Different distribution of breast ductal carcinoma in situ, ductal carcinoma in situ with microinvasion, and invasion breast cancer. World J Surg Oncol 10:262

#5 Seo YY, Yoo IR, Park SY et al (2017) Ductal carcinoma in situ and ductal carcinoma in situ with microinvasion: correlation of FDG uptake with histological and biological prognostic factors. Breast Cancer 24:353-361

#6 Wan ZB, Gao HY, Wei L et al (2018) Expression of estrogen receptor, progesterone receptor, human epidermal growth factor receptor 2, and Ki-67 in ductal carcinoma in situ (DCIS) and DCIS with microinvasion. Medicine (Baltimore) 97:e13055

#7 González LO, González-Reyes S, Junquera S et al (2010) Expression of metalloproteases and their inhibitors by tumor and stromal cells in ductal carcinoma in situ of the breast and their relationship with microinvasive events. J Cancer Res Clin Oncol 136:1313-21

#8 Ozkan-Gurdal S, Cabioglu N, Ozcinar B et al (2014) Factors predicting microinvasion in Ductal Carcinoma in situ. Asian Pac J Cancer Prev 15:55-60

#9 Okumura Y, Yamamoto Y, Zhang Z et al (2008) Identification of biomarkers in ductal carcinoma in situ of the breast with microinvasion. BMC Cancer 8:287

#10 Wang H, Lin J, Lai J et al (2019). Imaging features that distinguish pure ductal carcinoma *in situ* (DCIS) from DCIS with microinvasion. Mol Clin Oncol 11:313-319

#11 Costarelli L, Cianchetti E, Corsi F et al (2019). Microinvasive breast carcinoma: An analysis from ten Senonetwork Italia breast centres. Eur J Surg Oncol 45:147-152

#12 Hahn SY, Han BK, Ko EY et al (2013) MR features to suggest microinvasive ductal carcinoma of the breast: can it be differentiated from pure DCIS? Acta Radiol 54:742-8

#13 Meretoja TJ, Heikkilä PS, Salmenkivi K et al (2012) Outcome of patients with ductal carcinoma in situ and sentinel node biopsy. Ann Surg Oncol 19:2345-51

#14 Mori M, Tsugawa K, Yamauchi H et al (2013) Pathological assessment of microinvasive carcinoma of the breast. Breast Cancer 20:331-5

#15 Yao JJ, Zhan WW, Chen M et al (2015) Sonographic Features of Ductal Carcinoma In Situ of the Breast With Microinvasion: Correlation With Clinicopathologic Findings and Biomarkers. J Ultrasound Med 34:1761-8

#16 Allred DC, Harvey JM, Berardo M et al (1998) Prognostic and predictive factors in breast cancer by immunohistochemical analysis. Mod Pathol 11:155-68

#17 Wolff AC, Hammond ME, Hicks DG et al (2014) American Society of Clinical Oncology; College of American Pathologists. Recommendations for human epidermal growth factor receptor 2 testing in breast cancer: American Society of Clinical Oncology/College of American Pathologists clinical practice guideline update. Arch Pathol Lab Med 138:241-56

**Supplementary Table S5.**

Summary of total cases, median cases, and median follow-up term in our meta-analysis

|  | Risk group | Reference group | Number of total studies (n) | Risk group (DCIS-Mi) | | Reference group (DCIS) | |
| --- | --- | --- | --- | --- | --- | --- | --- |
|  |  |  |  | Total cases (n) | Median cases  (n [range]) | Total cases (n) | Median cases  (n [range]) |
| DFS | DCIS-Mi | DCIS | 6 | 744 | 108 [67–242] | 2,381 | 405 [271–543] |
| OS | DCIS-Mi | DCIS | 3 | 207 | 72 [51–84] | 1,018 | 432 [154–543] |
| LRFS | DCIS-Mi | DCIS | 3 | 577 | 84 [72–421] | 3,564 | 543 [321–2700] |
| DMFS | DCIS-Mi | DCIS | 3 | 577 | 84 [72–421] | 3,564 | 543 [321–2700] |

Abbreviations: DCIS-Mi, ductal carcinoma *in situ* with microinvasion; DCIS, ductal carcinoma *in situ*; DFS, disease-free survival; DMFS, distant metastasis-free survival; LRFS, loco-regional recurrence-free survival; OS, overall survival.

**Supplementary Table S6.**

The rate of each clinicopathological characteristic between DCIS-Mi and DCIS group in selected articles

|  | The number of articles | DCIS-Mi group | DCIS group | *p* value |
| --- | --- | --- | --- | --- |
|  |  | The median rate:  % (range) | The median rate:  % (range) |  |
| Premenopausal status | 9 | 53.7 (25.0–69.8) | 56.9 (36.0–71.2) | 0.214 |
| Tumor size (>2cm) | 6 | 50.7 (27.2–72.4) | 43.0 (21.9–52.5) | **0.046** |
| Axillary LN metastasis | 16 | 7.37 (2.17–20.8) | 0.73 (0–6.85) | **< 0.001** |
| Comedo necrosis | 10 | 49.9 (16.7–78.4) | 25.4 (3.2–53.7) | **0.005** |
| Nuclear grade 3 | 15 | 67.4 (10.7–84.2) | 37.1 (3.85–47.5) | **0.001** |
| ER-positivity | 6 | 49.2 (43.8–77.2) | 75.3 (69.9–91.5) | **0.028** |
| PR-positivity | 6 | 42.2 (23.6–69.6) | 63.8 (53.7–80.9) | **0.028** |
| HER2-positivity | 7 | 56.4 (39.1–73.7) | 29.5 (18.8–45.6) | **0.018** |
| BCS | 13 | 42.1 (5.43–100) | 58.8 (11.8–100) | 0.05 |
| Adjuvant HT | 8 | 33.9 (7.12–62.2) | 23.7 (1.04–60.5) | 0.069 |
| Adjuvant CT | 5 | 13.4 (5.71–19.0) | 1.86 (0–5.1) | **0.043** |
| Adjuvant HER2 targeted therapy | 2 | 3.21 (2.86–3.57) | 0 (0) | 0.180 |
| Adjuvant radiotherapy | 7 | 43.9 (34.5–83.5) | 50.6 (15.4–75.4) | 0.398 |

Abbreviations: BCS, breast conserving therapy; CT, chemotherapy; DCIS, ductal carcinoma *in situ*; DCIS-Mi, ductal carcinoma *in situ* with microinvasion; ER, estrogen receptor; HER2, human epidermal growth factor receptor 2; HT, hormone therapy; LN, lymph node; PR, progesterone receptor

Bold letters indicate statistical significance.

**Supplementary Table S7.**

Meta-regression analysis results of the relationship between hazard ratios for DFS analysis and the rates of each clinicopathological factor in the DCIS-Mi group

|  | Regression coefficient | SE | 95% CI | *p* value |
| --- | --- | --- | --- | --- |
| Premenopausal status | -0.0334 | 0.028 | -0.0884 to 0.0216 | 0.234 |
| Tumor size > 2cm | -0.0485 | 0.0376 | -0.122 to 0.0252 | 0.197 |
| Axillary LN metastasis | 0.072 | 0.124 | -0.17 to 0.314 | 0.560 |
| Nuclear grade 3 | -0.00877 | 0.00916 | -0.0267 to 0.00918 | 0.338 |
| ER-positivity | 0.000801 | 0.0493 | -0.0958 to 0.0974 | 0.987 |
| PR-positivity | -0.0154 | 0.0299 | -0.074 to 0.0431 | 0.606 |
| HER2-positivity | -0.0736 | 0.0573 | -0.186 to 0.0386 | 0.198 |
| Adjuvant HT | 0.0231 | 0.0467 | -0.0685 to 0.115 | 0.621 |
| Adjuvant CT | 0.000628 | 0.119 | -0.233 to 0.234 | 0.996 |
| Adjuvant radiotherapy | -0.00391 | 0.74 | -1.46 to 1.45 | 0.996 |

Abbreviations: CI, confidence interval; CT, chemotherapy; DCIS-Mi, ductal carcinoma in situ with microinvasion; DFS, disease-free survival; ER, estrogen receptor; HER2, human epidermal growth factor receptor 2; HT, hormone therapy; LN, lymph node; PR, progesterone receptor; SE, standard error.

**Supplementary Figure S1.**

Screening process by the 2^nd^ reviewer

**Supplementary Figure S2.** Risk of bias using RoBANS tool.

(A) Risk of bias summary

(B) Risk of bias graph

(C) κ value according to each category

(B)

(A)

| Risk of bias assessment | κ value |
| --- | --- |
| The selection of participants | 1.00 |
| Confounding variables | 1.00 |
| Measurement of exposure | 1.00 |
| Blinding of outcome assessments | 1.00 |
| Incomplete outcome data | 0.44 |
| Selective outcome reporting | 1.00 |

(C)

**Supplementary Figure S3.** Forest plots comparing survival in the subgroup analysis based on risk of bias assessment

Abbreviations: DCIS, ductal carcinoma *in situ*; DCIS-Mi, ductal carcinoma *in situ* with microinvasion; DFS, disease-free survival; OS, overall survival.

1. Comparison of DFS between DCIS-Mi group and DCIS group
2. Comparison of OS between DCIS-Mi group and DCIS group

**Supplementary Figure S4.** Forest plots comparing each clinicopathological characteristic on DFS for patients with DCIS-Mi and no adjuvant treatment

Abbreviations: DCIS-Mi, ductal carcinoma *in situ* with microinvasion; DFS, disease-free survival; ER, estrogen receptor; PR, progesterone receptor; HER2, human epidermal growth factor receptor 2

1. Comparison of DFS between patients aged <50 years (risk) and ≥50 years (reference)
2. Comparison of DFS between ER-positivity (risk) and ER-negativity (reference)
3. Comparison of DFS between PR-positivity (risk) and PR-negativity (reference)
4. Comparison of DFS between HER2-positivity (risk) and HER2-negativity (reference)

**Supplementary Figure S5.**

Funnel plots for each meta-analysis

1. Disease-free survival (DFS) analysis
2. Overall survival (OS) analysis

(A)


(B)
